# Supplementary material for: Fission yeast RPA–TERT–Tpz1TPP1 complex promotes telomere extension and suppresses telomere recombination
Source: bioRxiv. 2026 Jul 28:2026.04.26.720725. Preprint. [Version 4] doi: 10.64898/2026.04.26.720725 (PMC13419711; doi:10.64898/2026.04.26.720725)

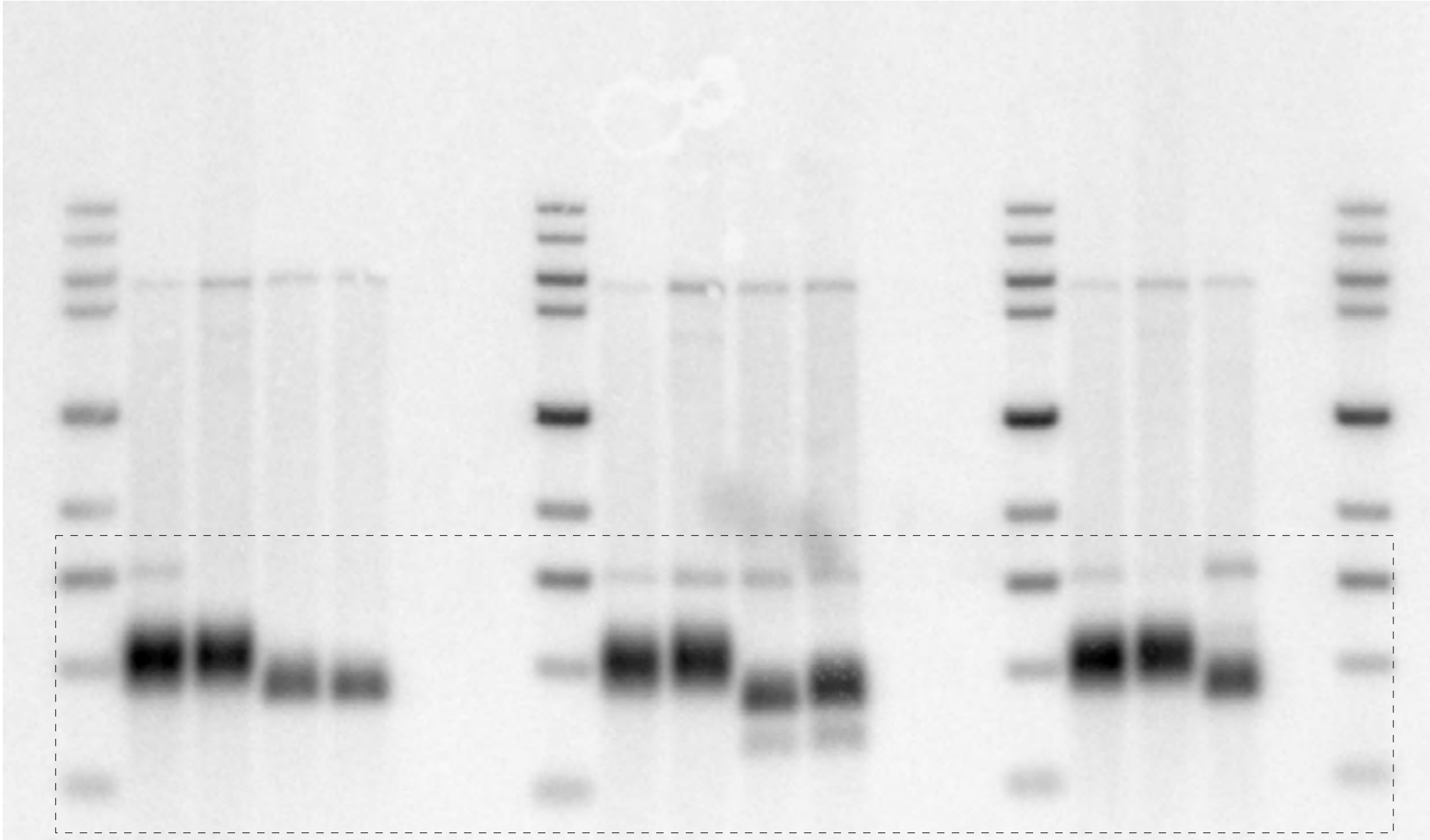

Fig 1B

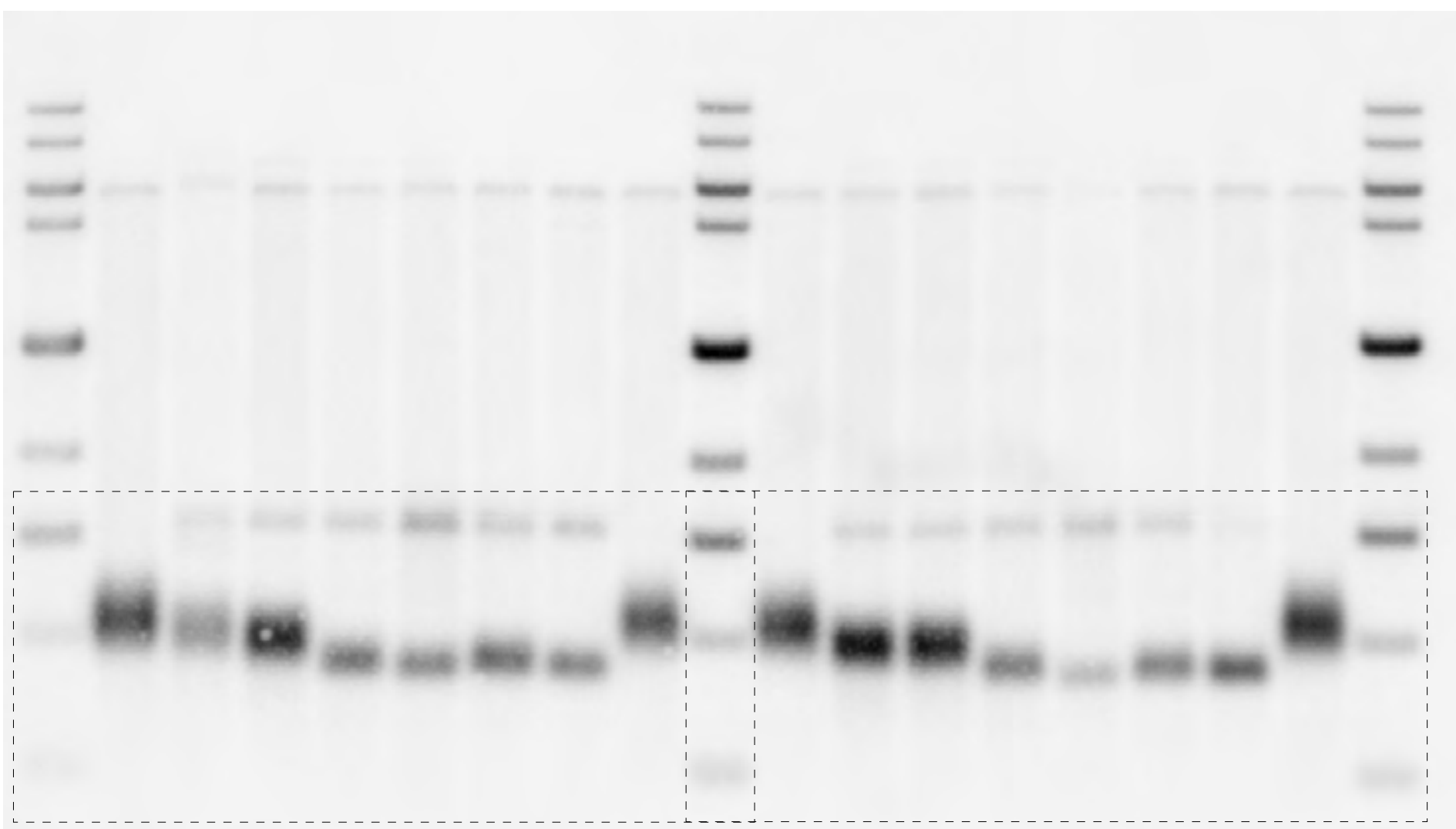

Fig S2B

Fig 1C

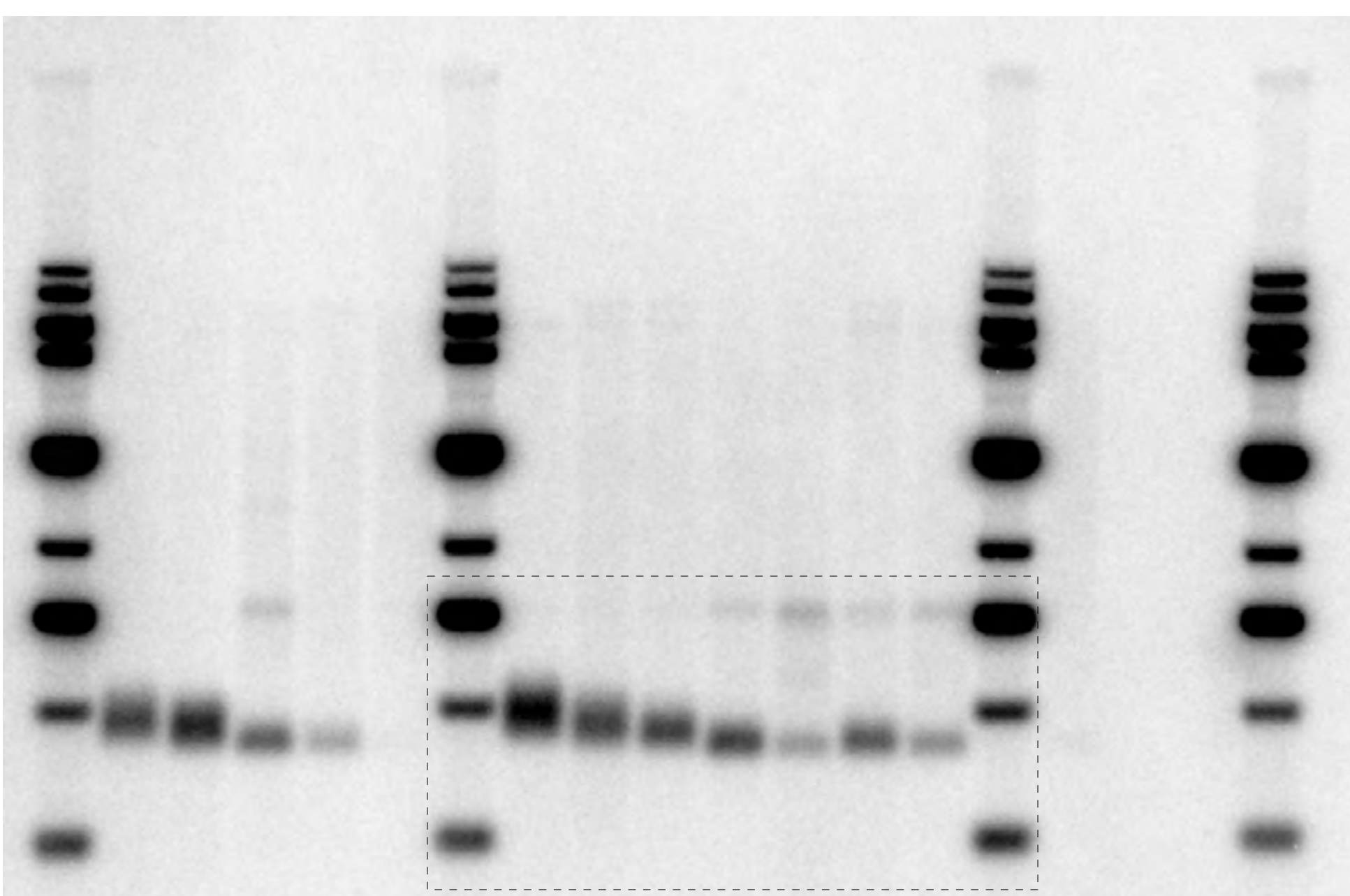

Fig 1D

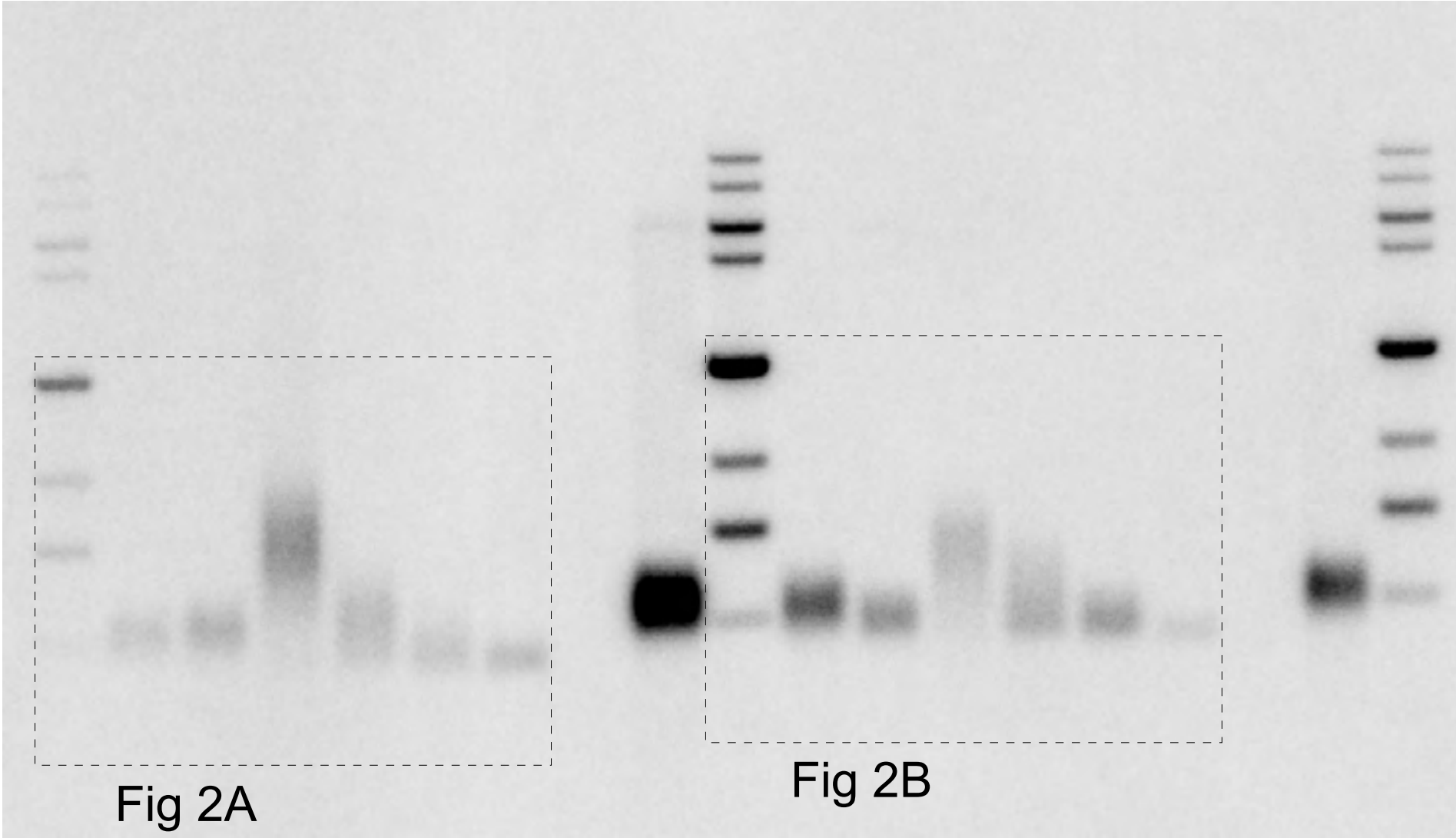
Fig 2A
Fig 2B

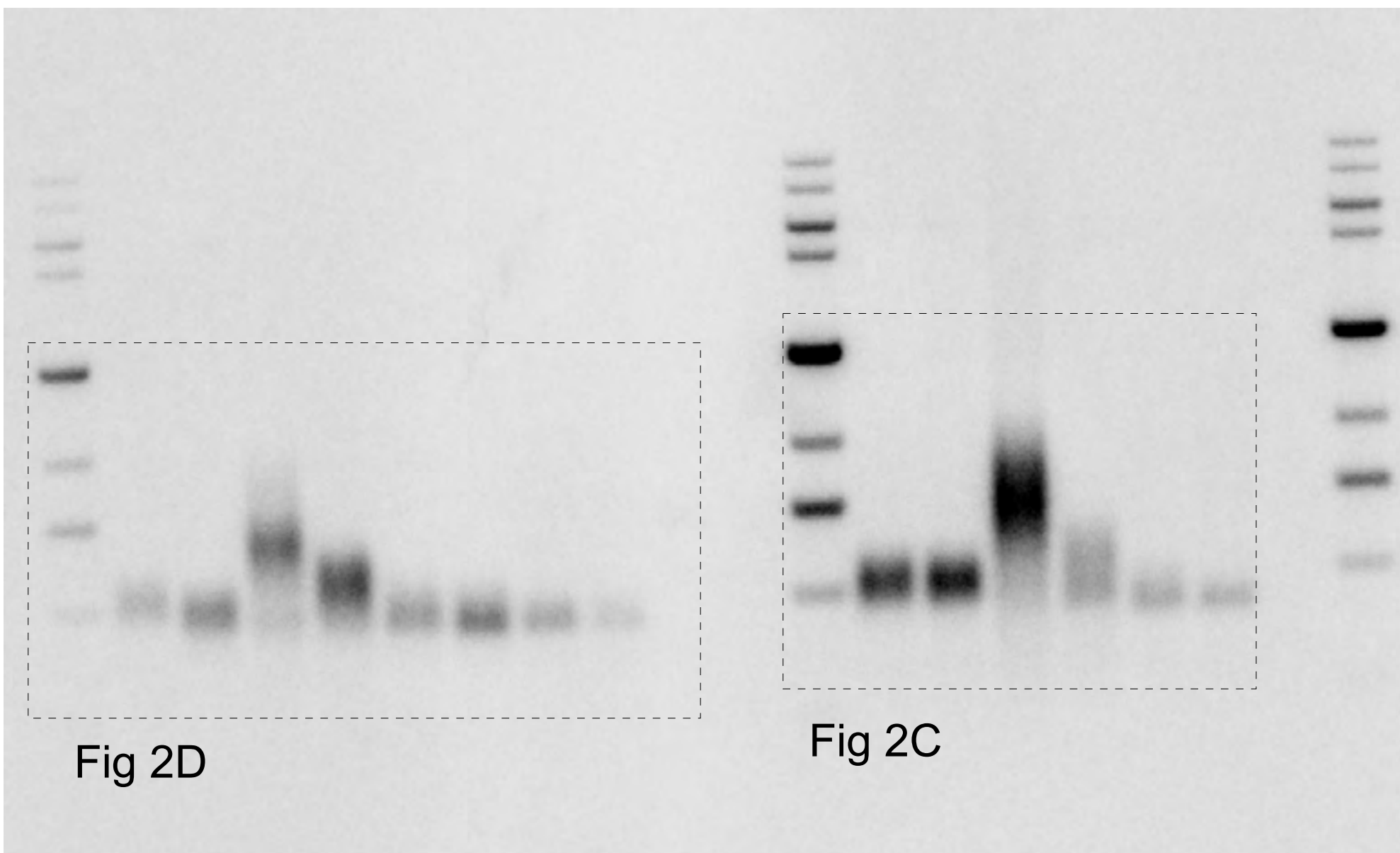
Fig 2D
Fig 2C

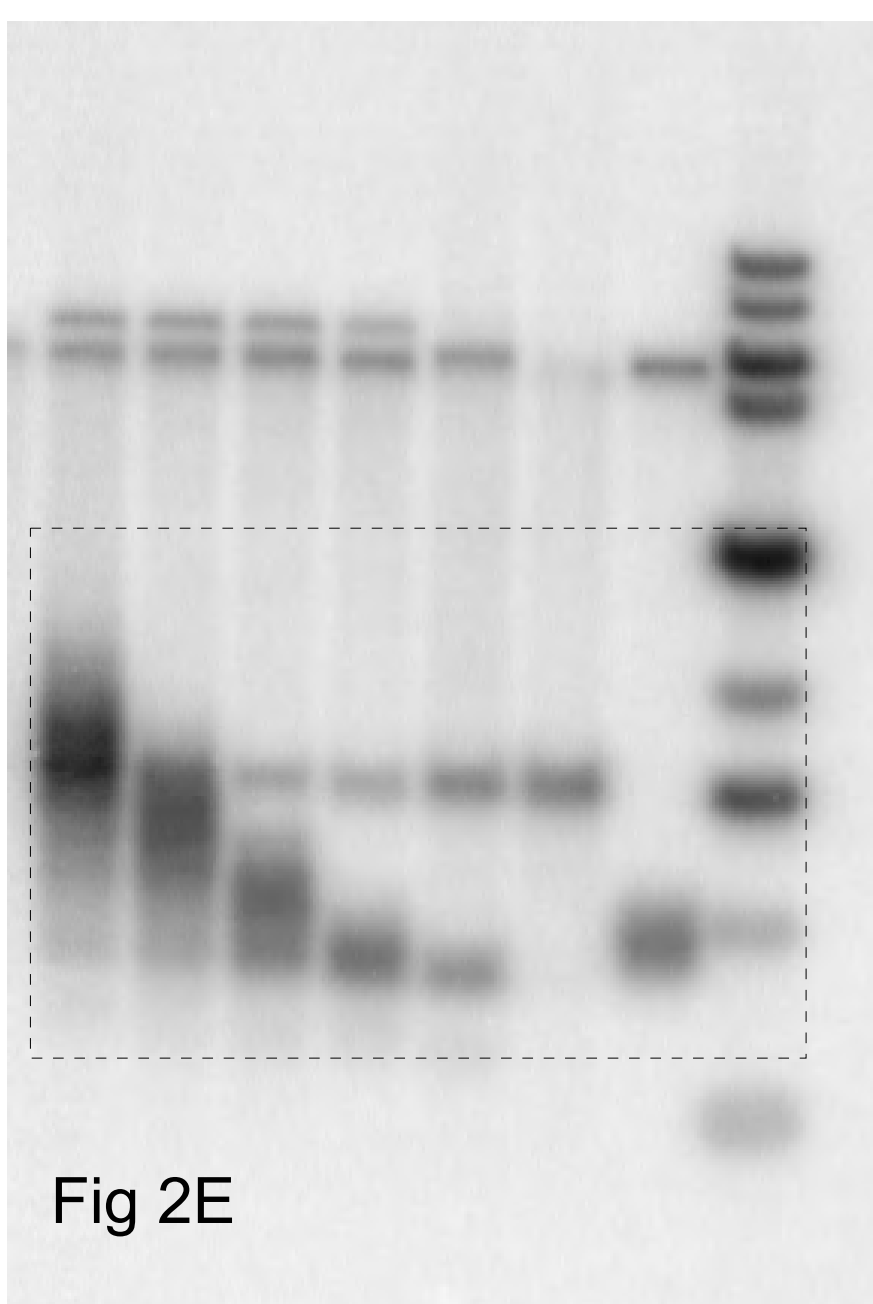
Fig 2E

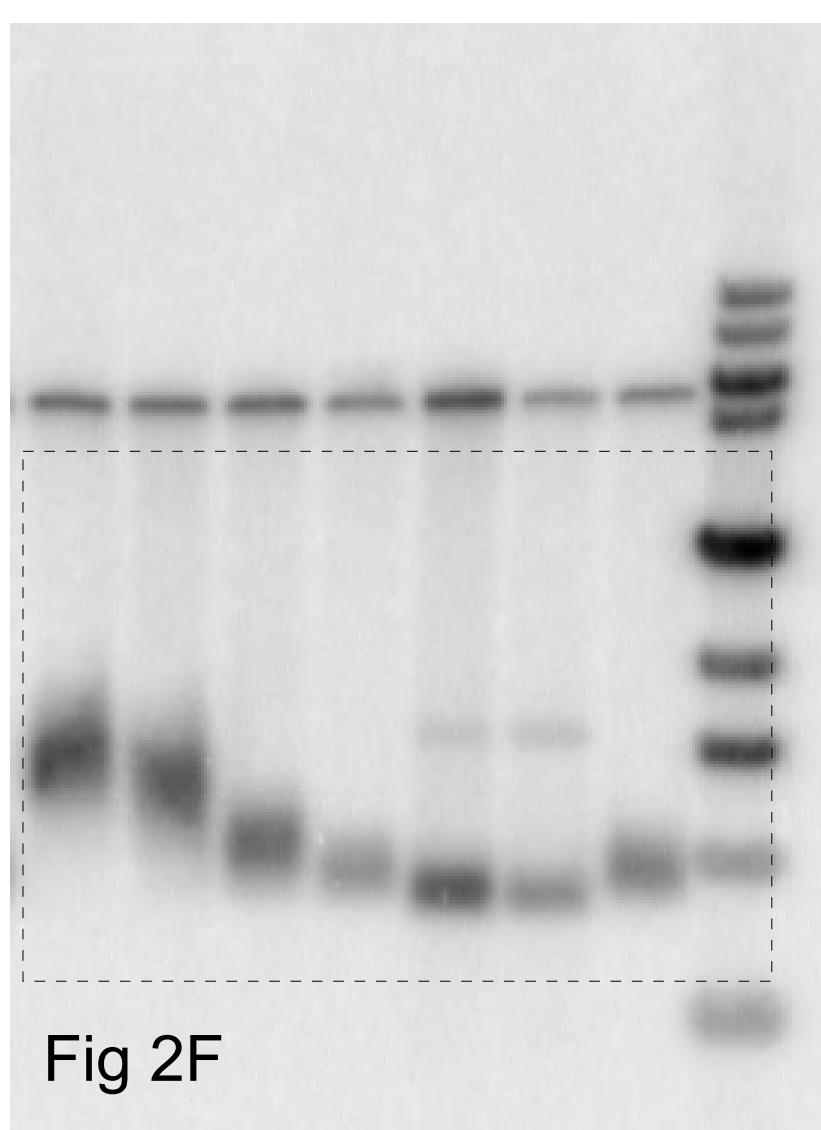
Fig 2F

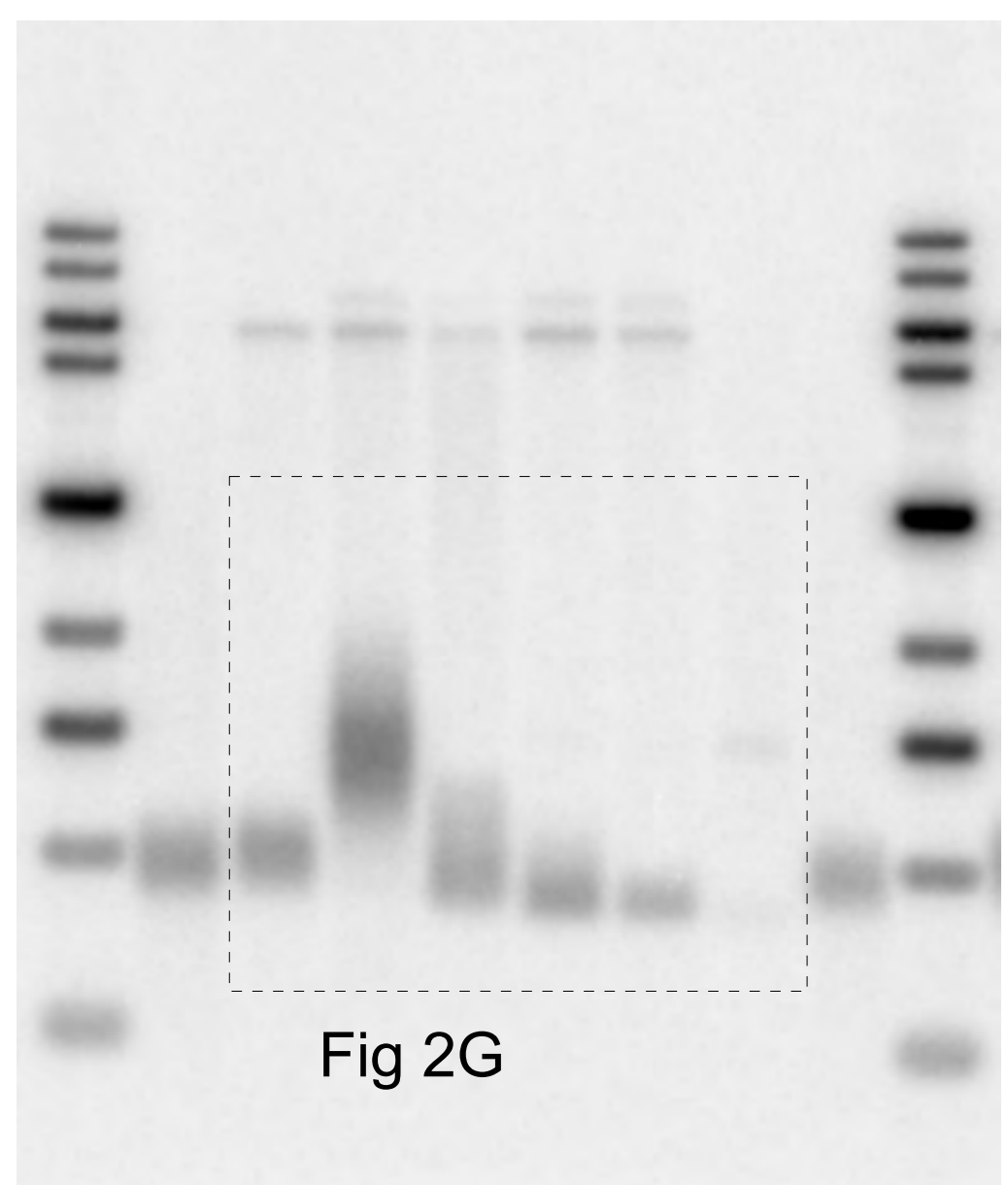
Fig 2G

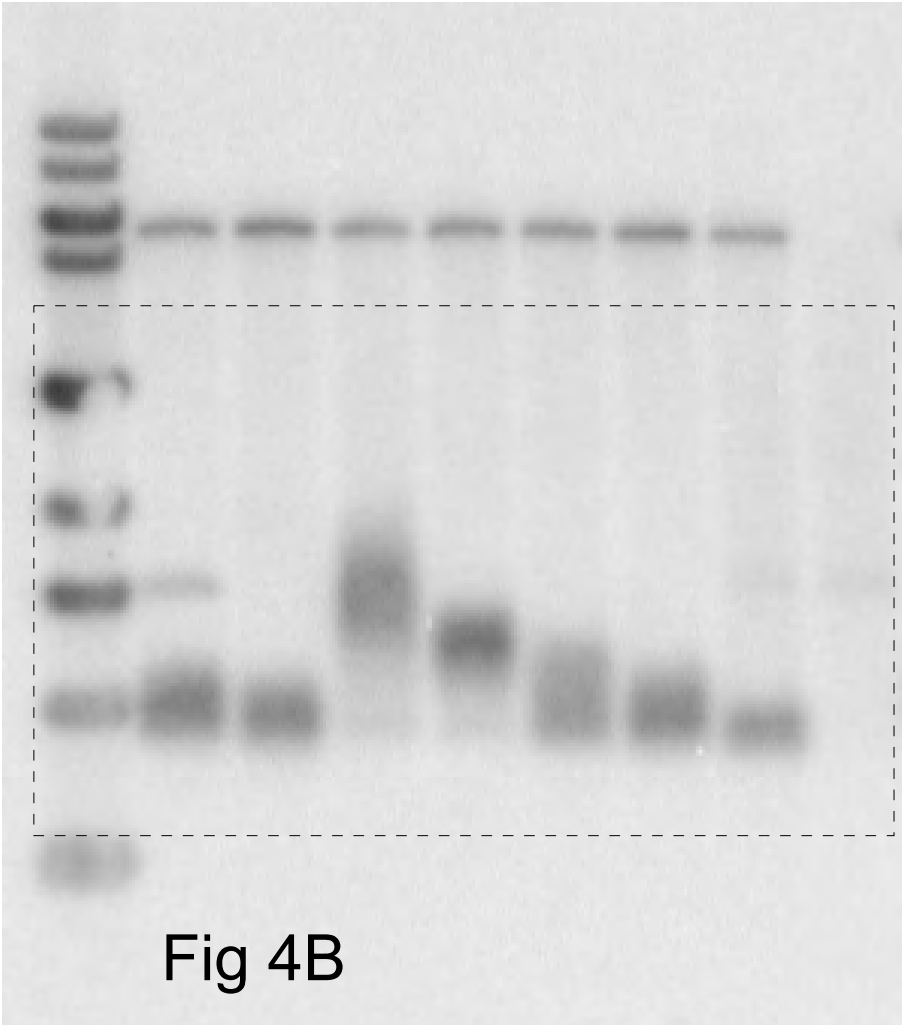
Fig 4B

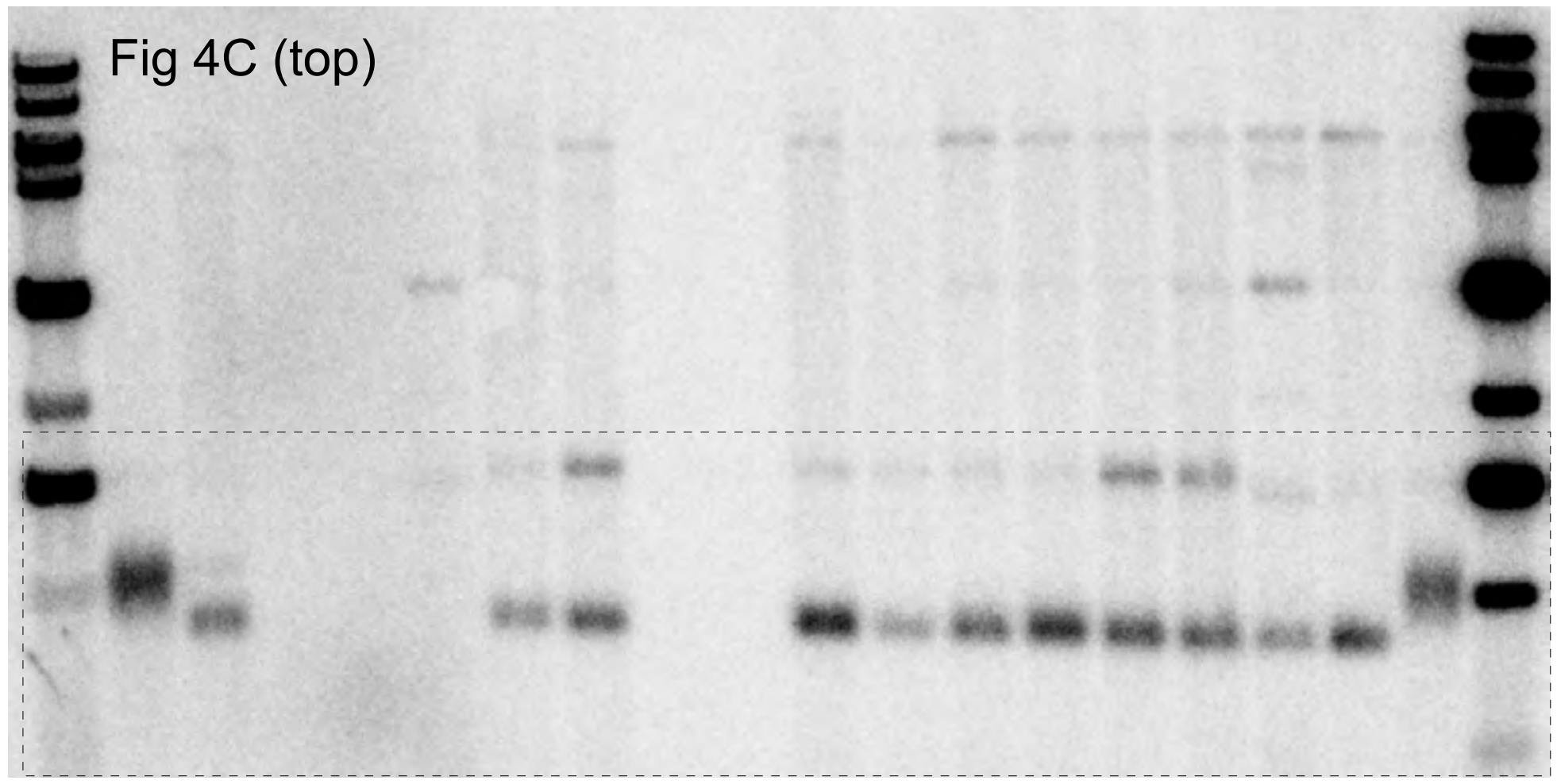
Fig 4C (top)

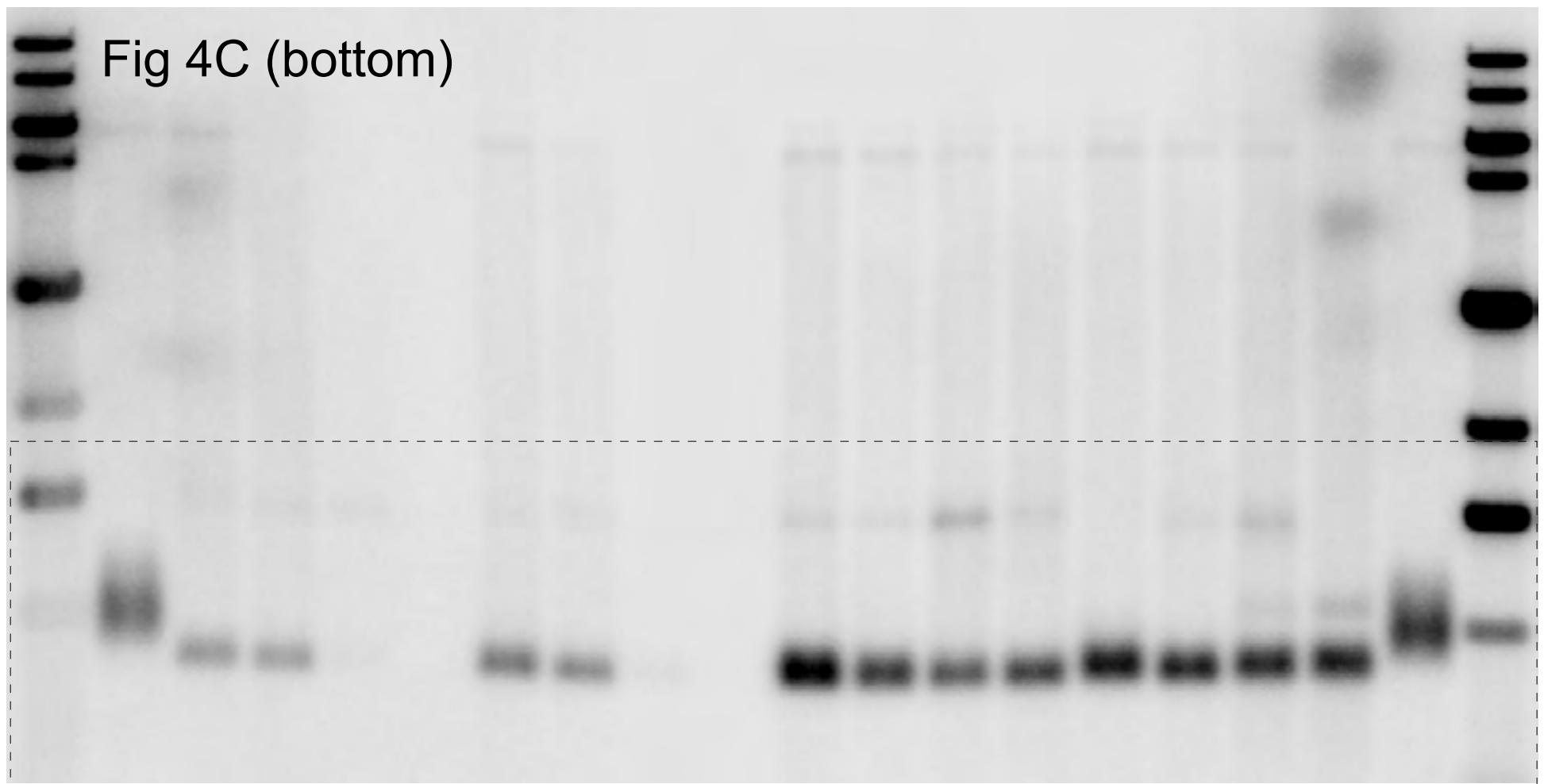
Fig 4C (bottom)

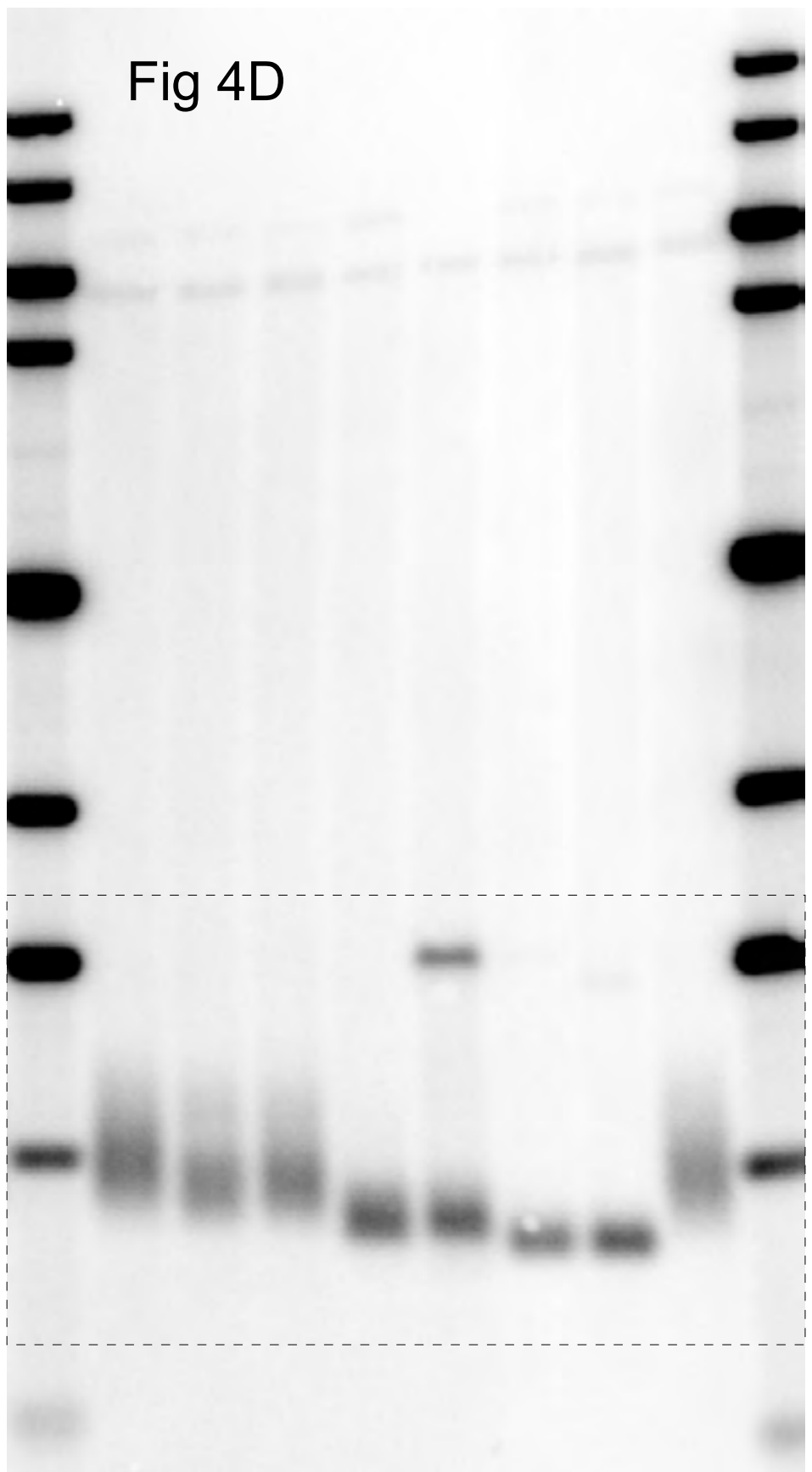
Fig 4D

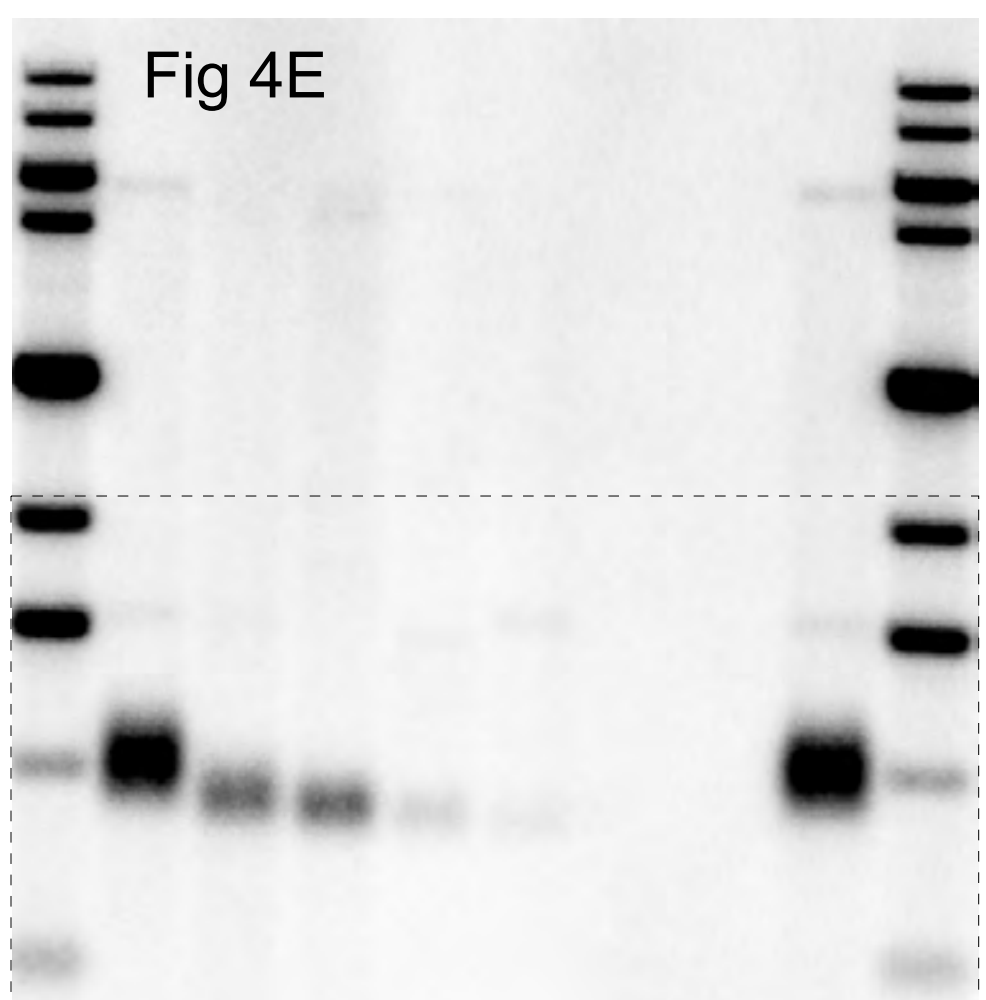
Fig 4E

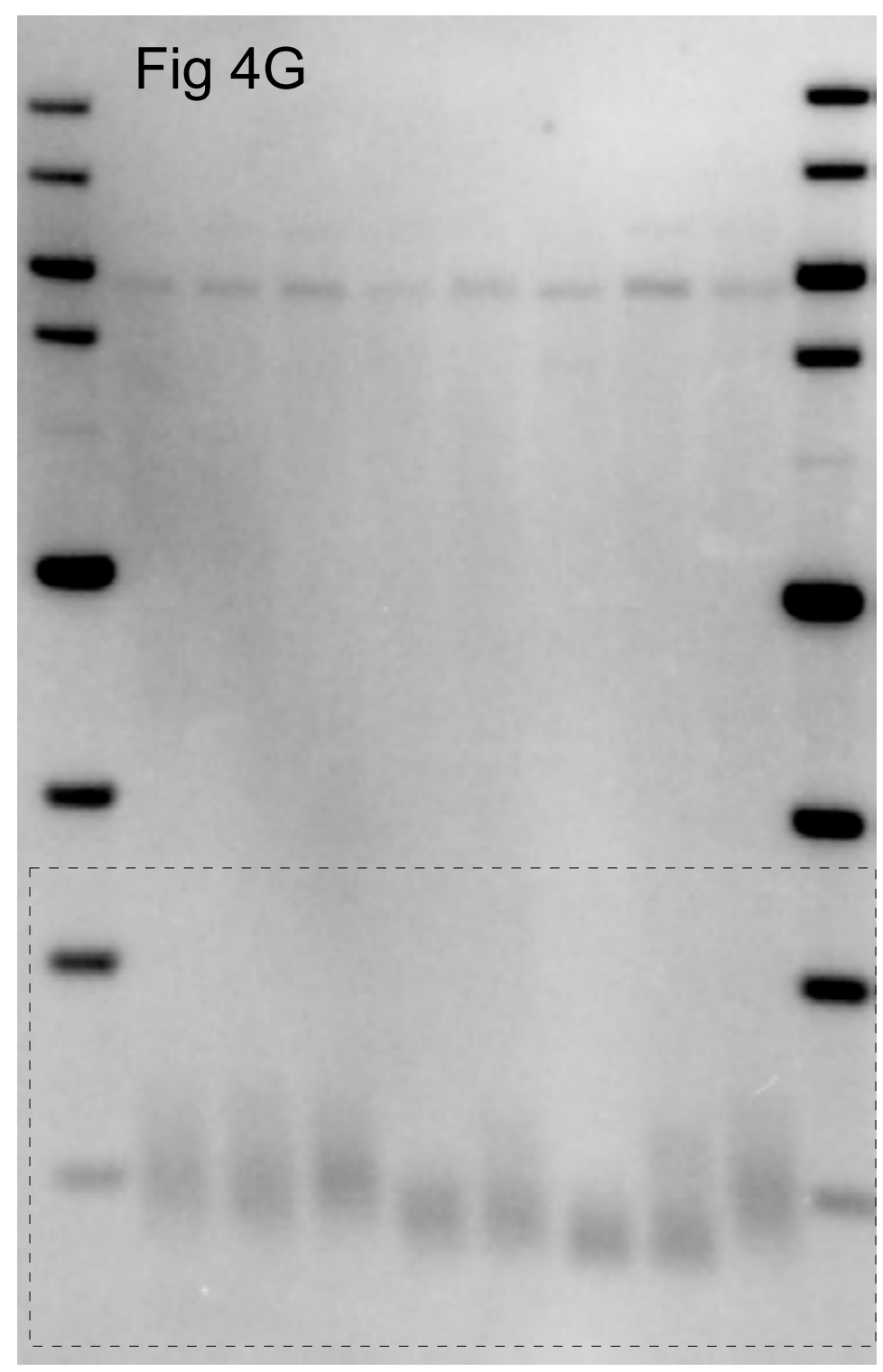
Fig 4G

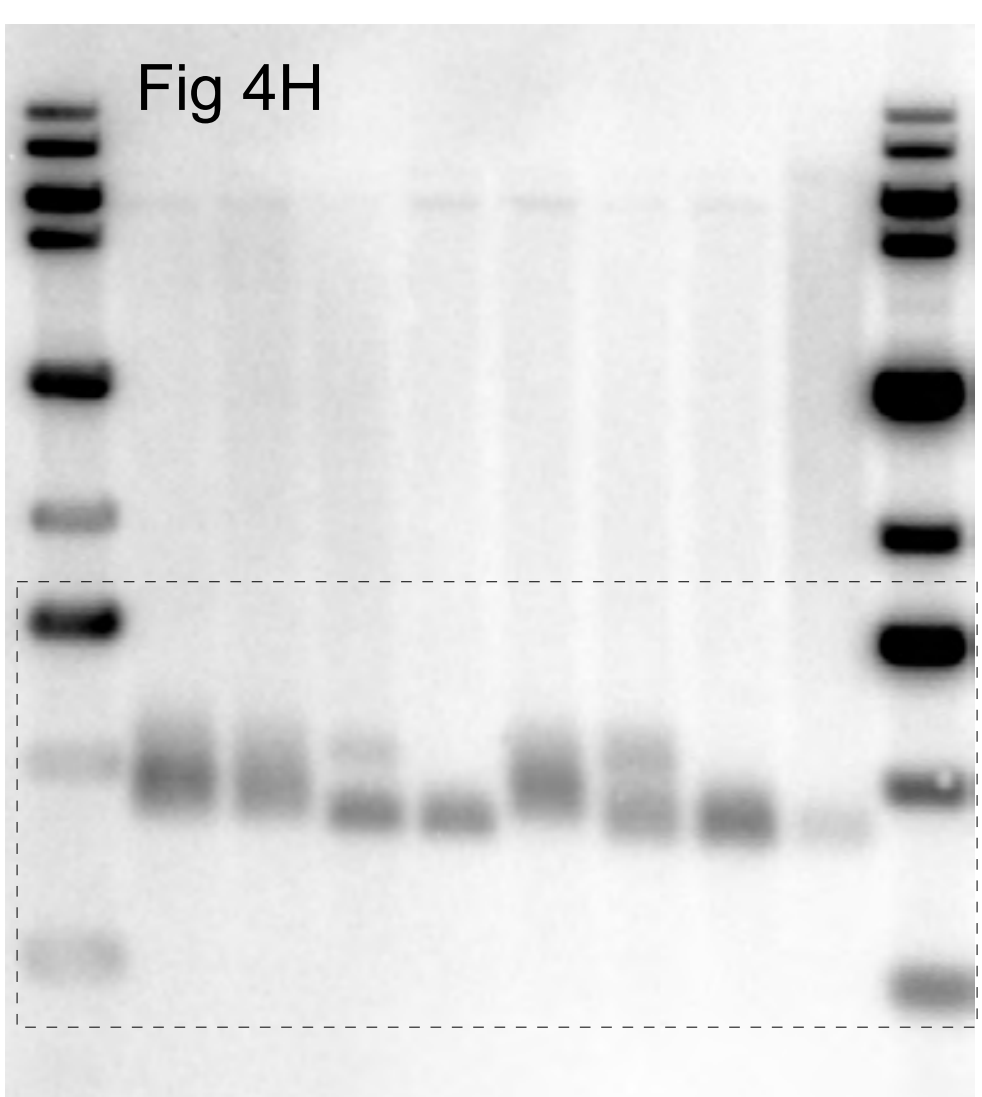
Fig 4H

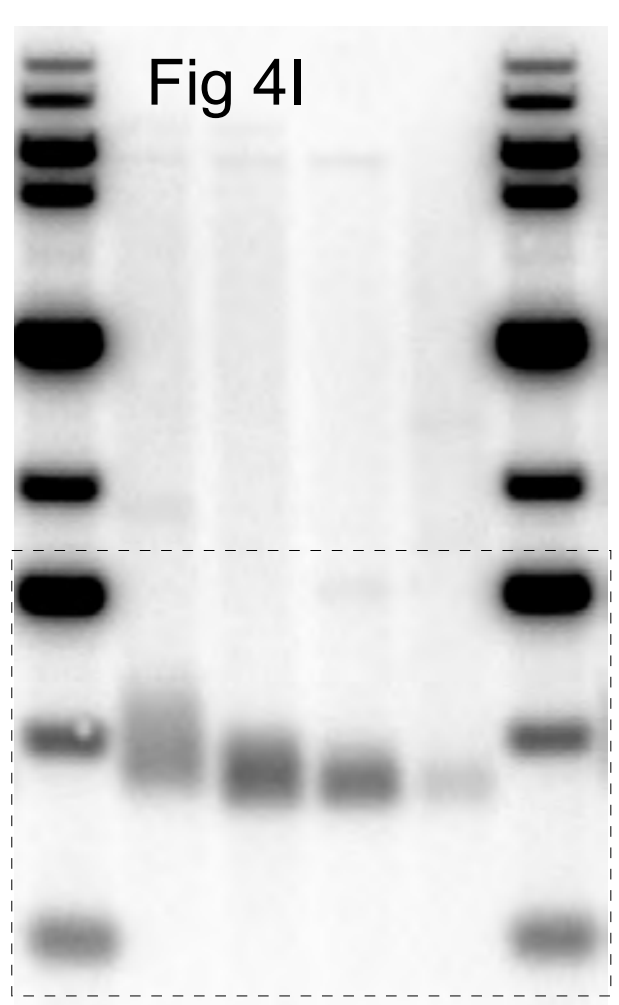
Fig 4I

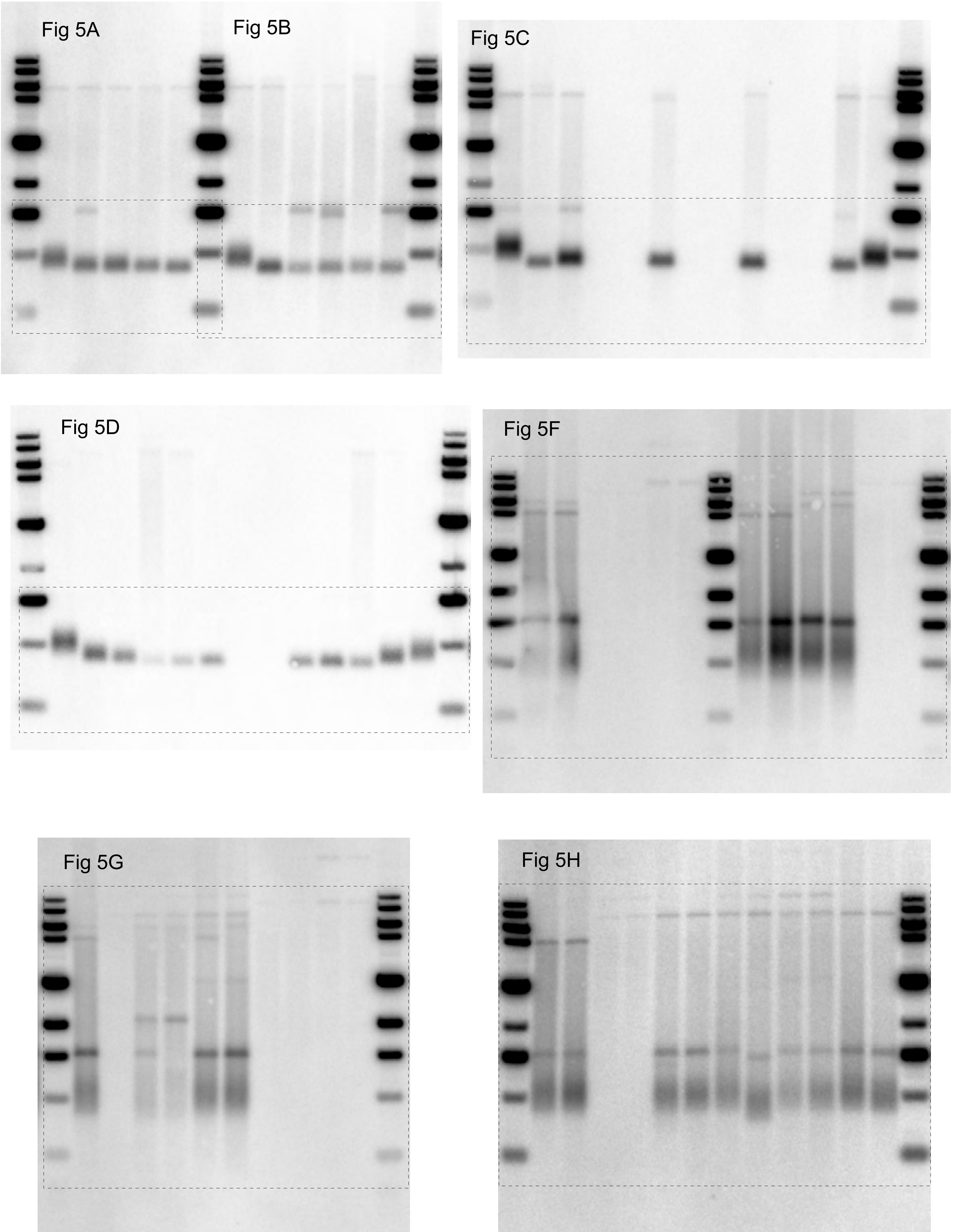
Fig 5A
Fig 5B
Fig 5C
Fig 5D
Fig 5F
Fig 5G
Fig 5H

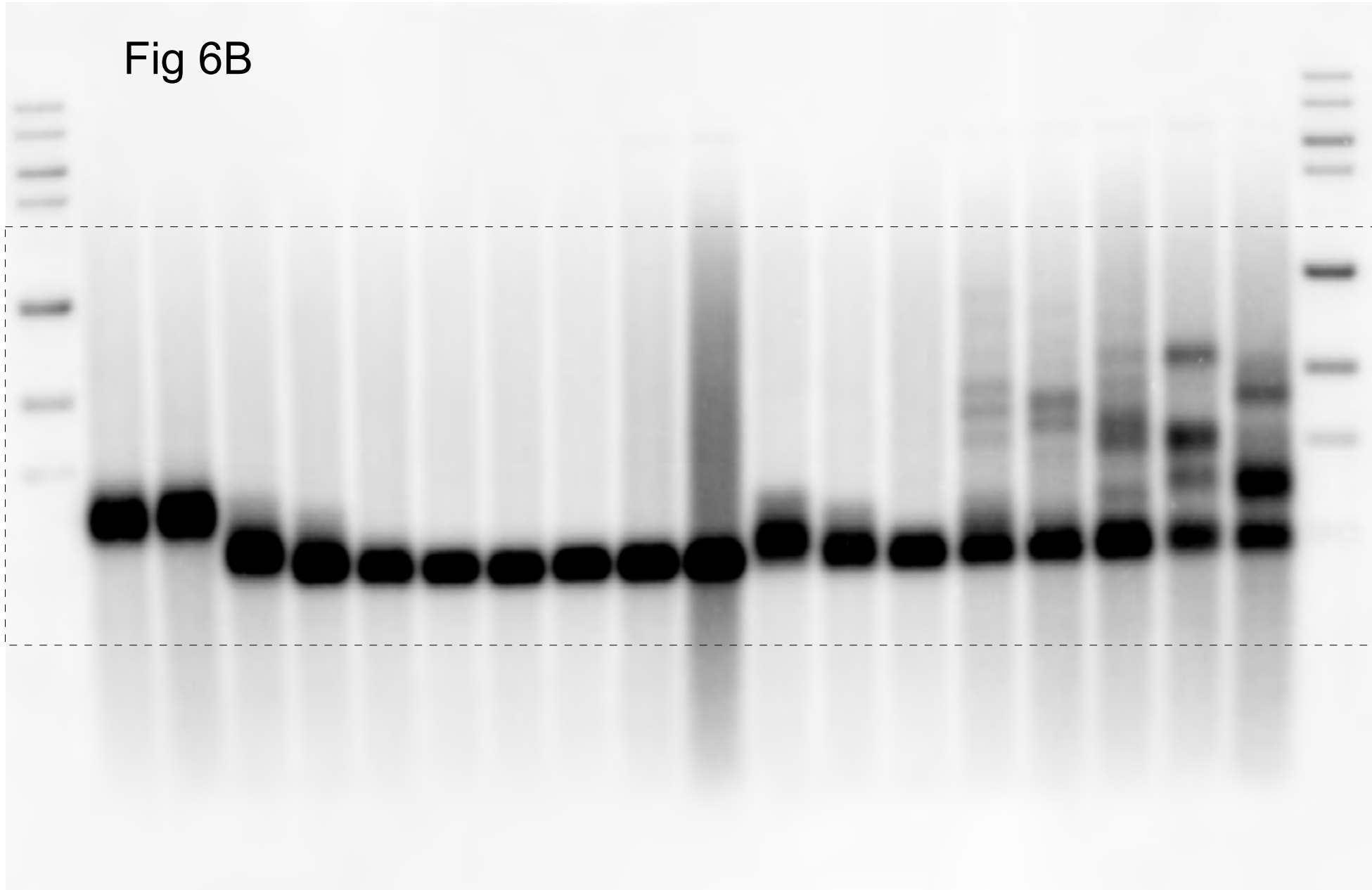

Fig 6C

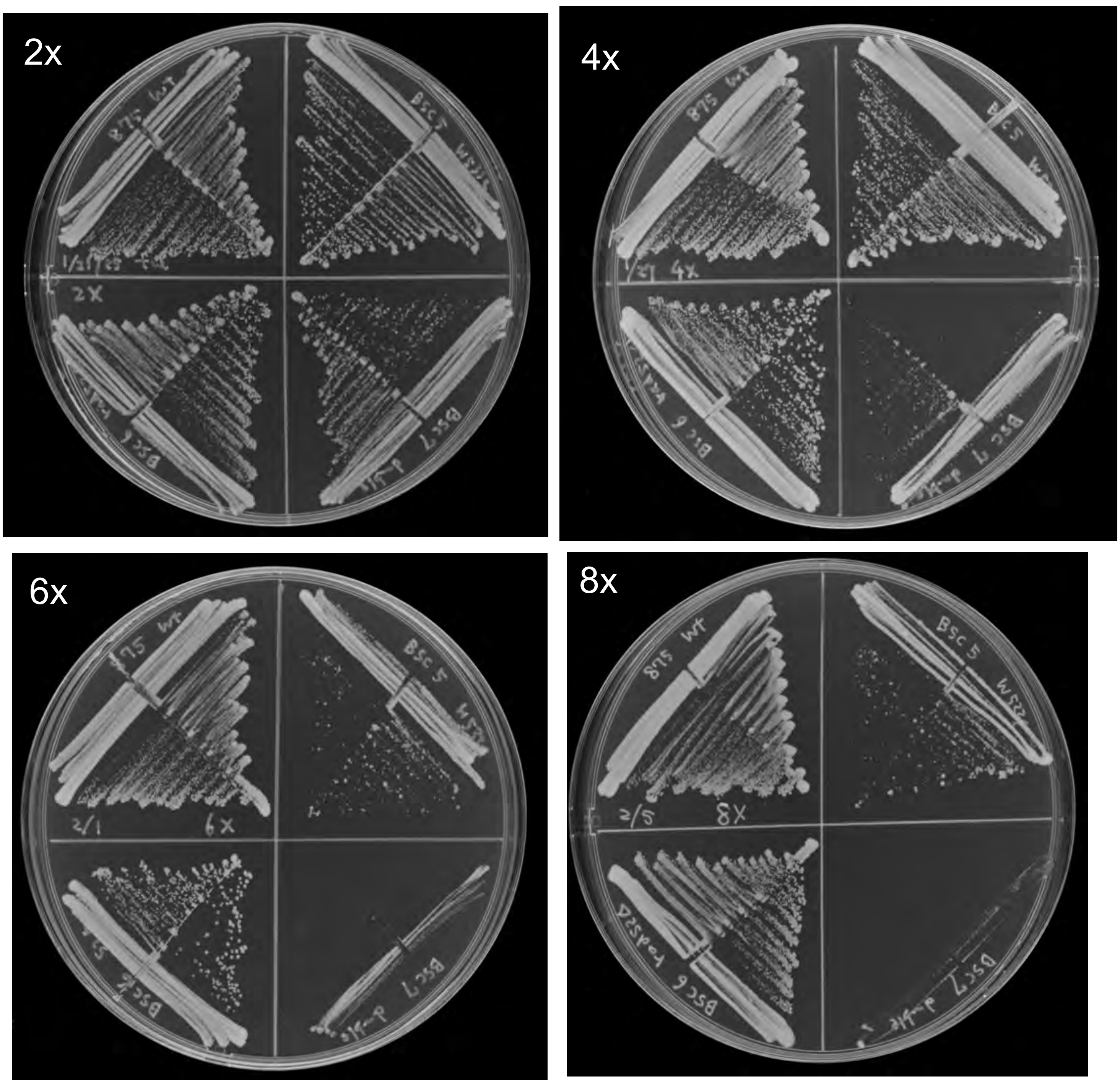

Fig 6E

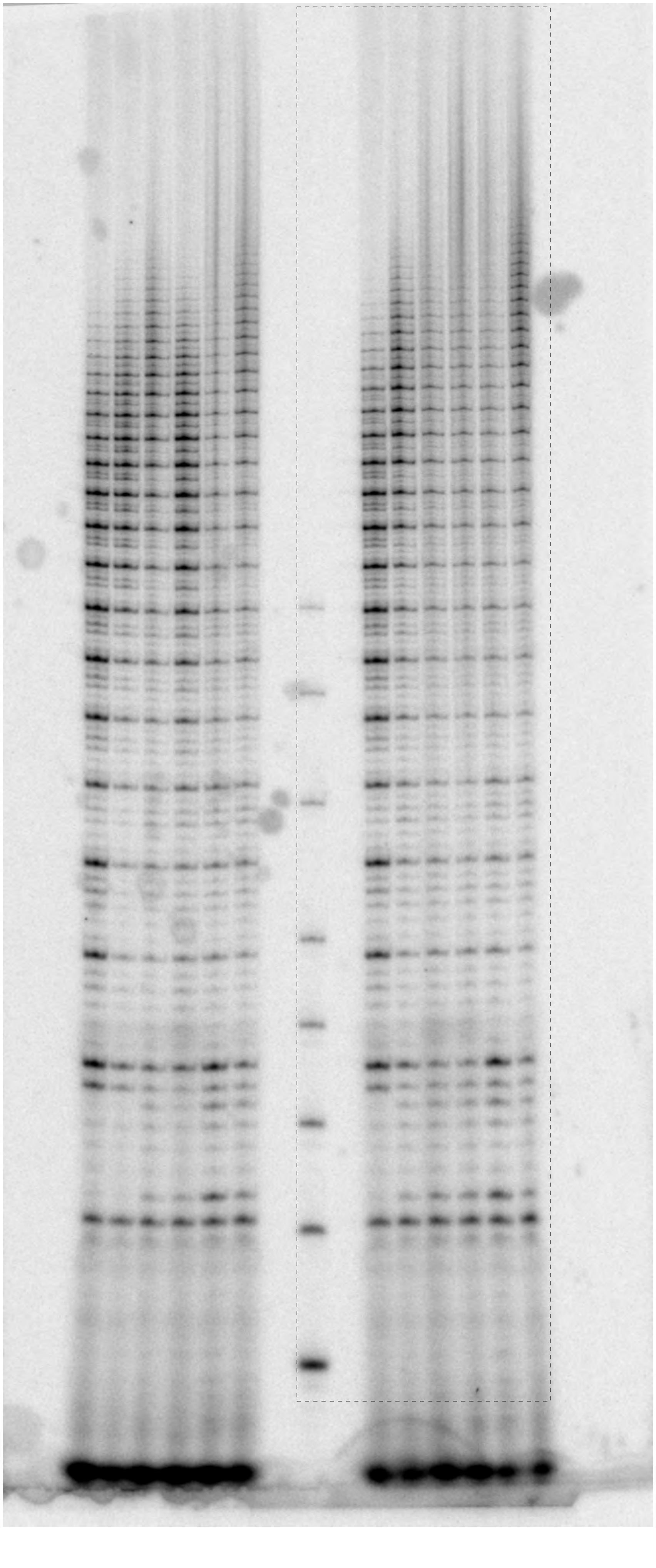

Fig S1B

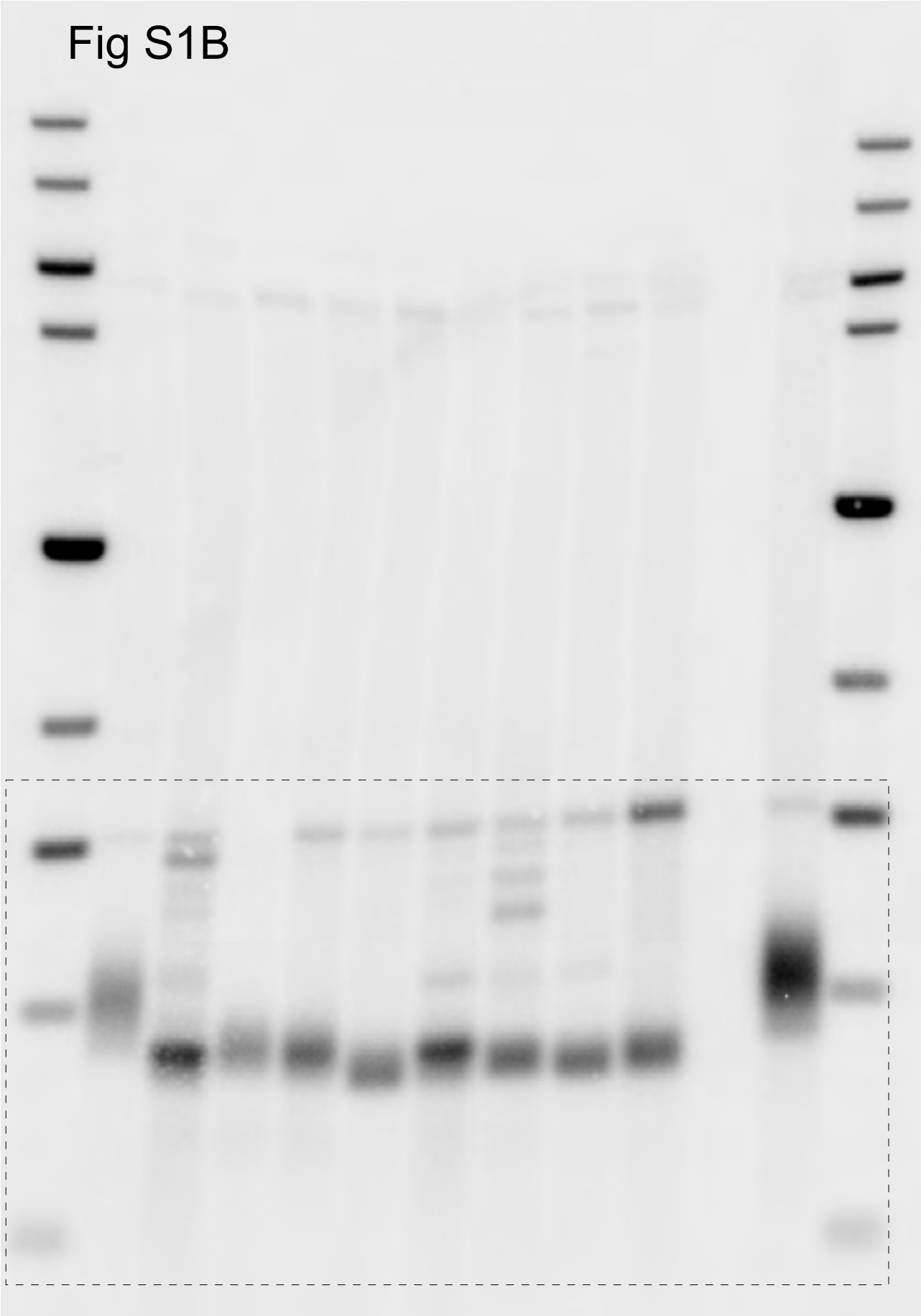

Fig S1D

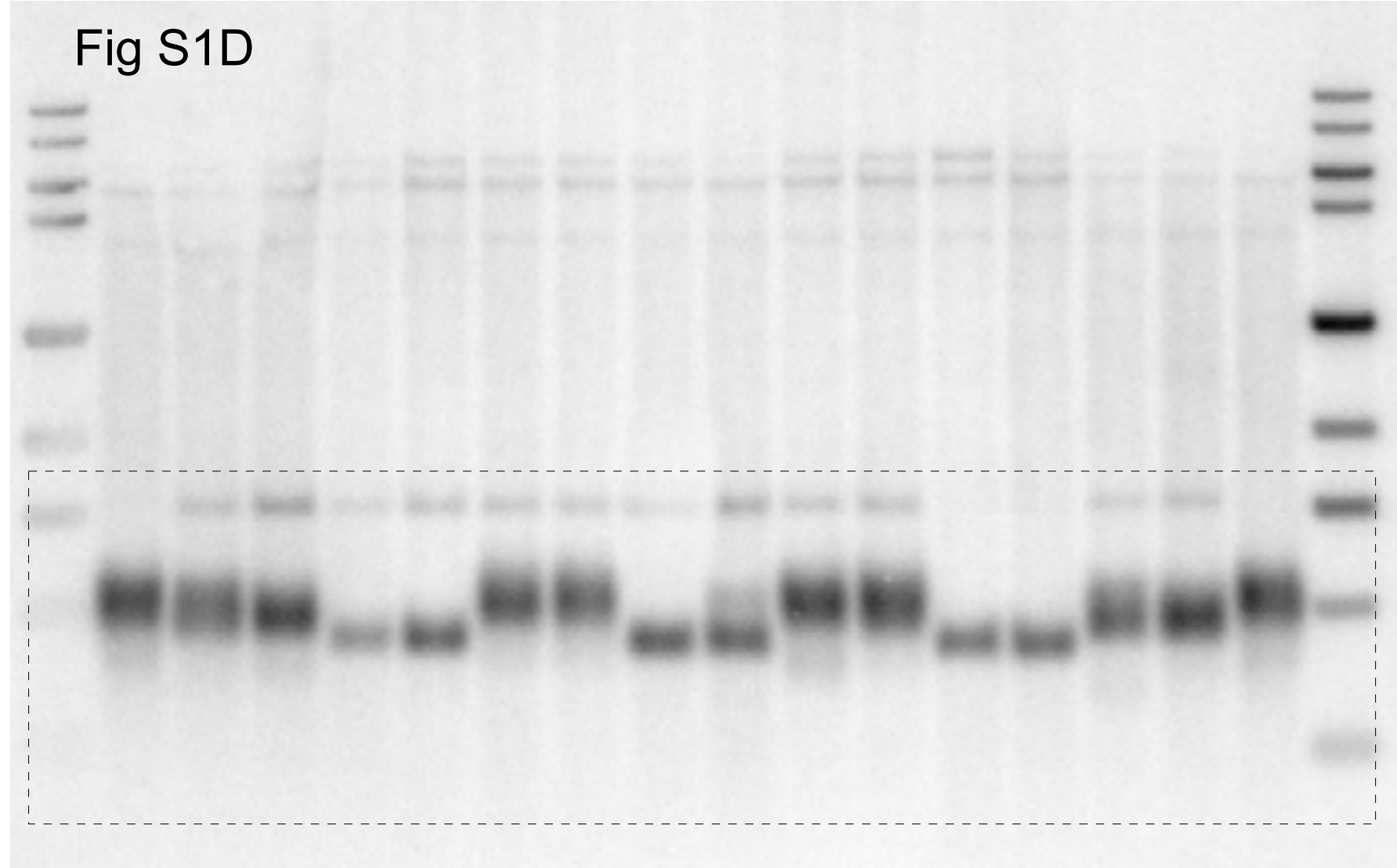

Fig S1D

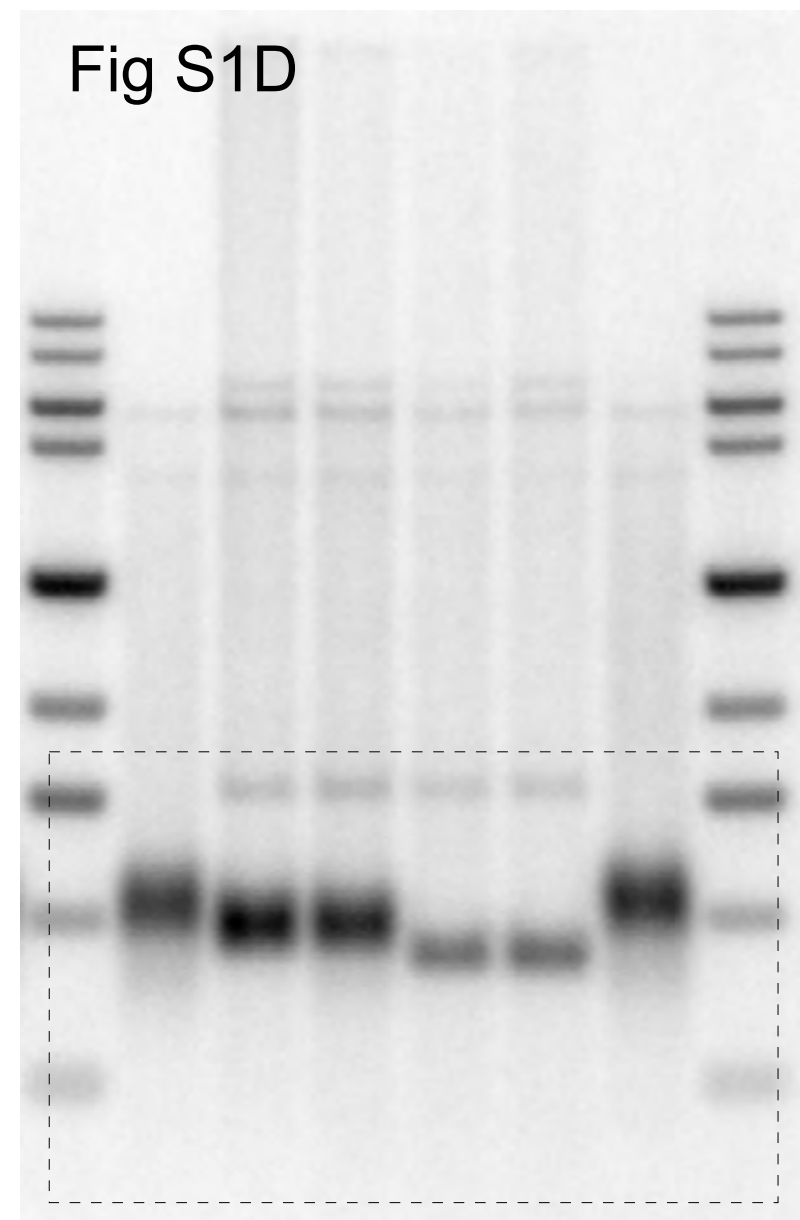

Fig S1C

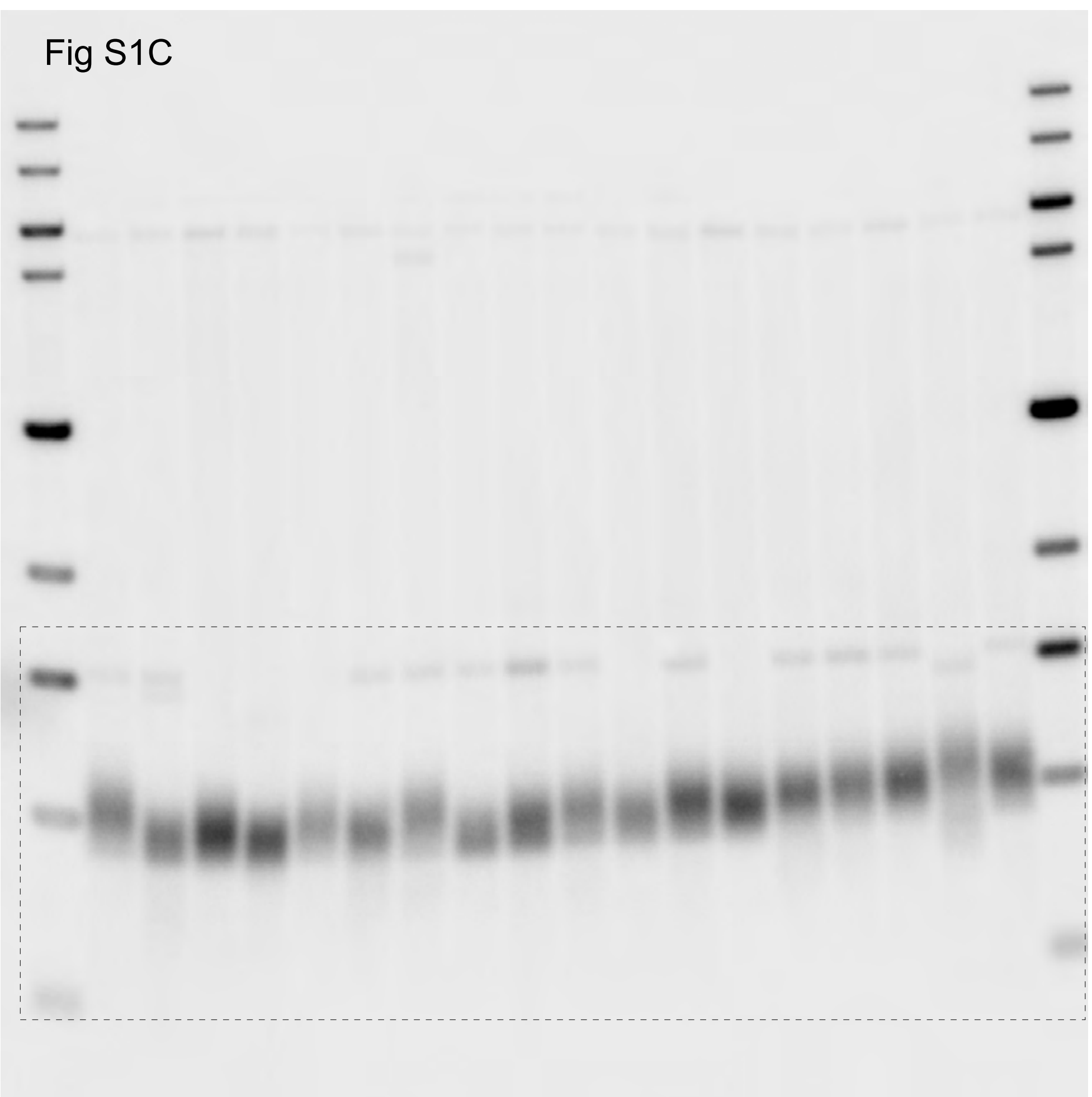

Fig S1E

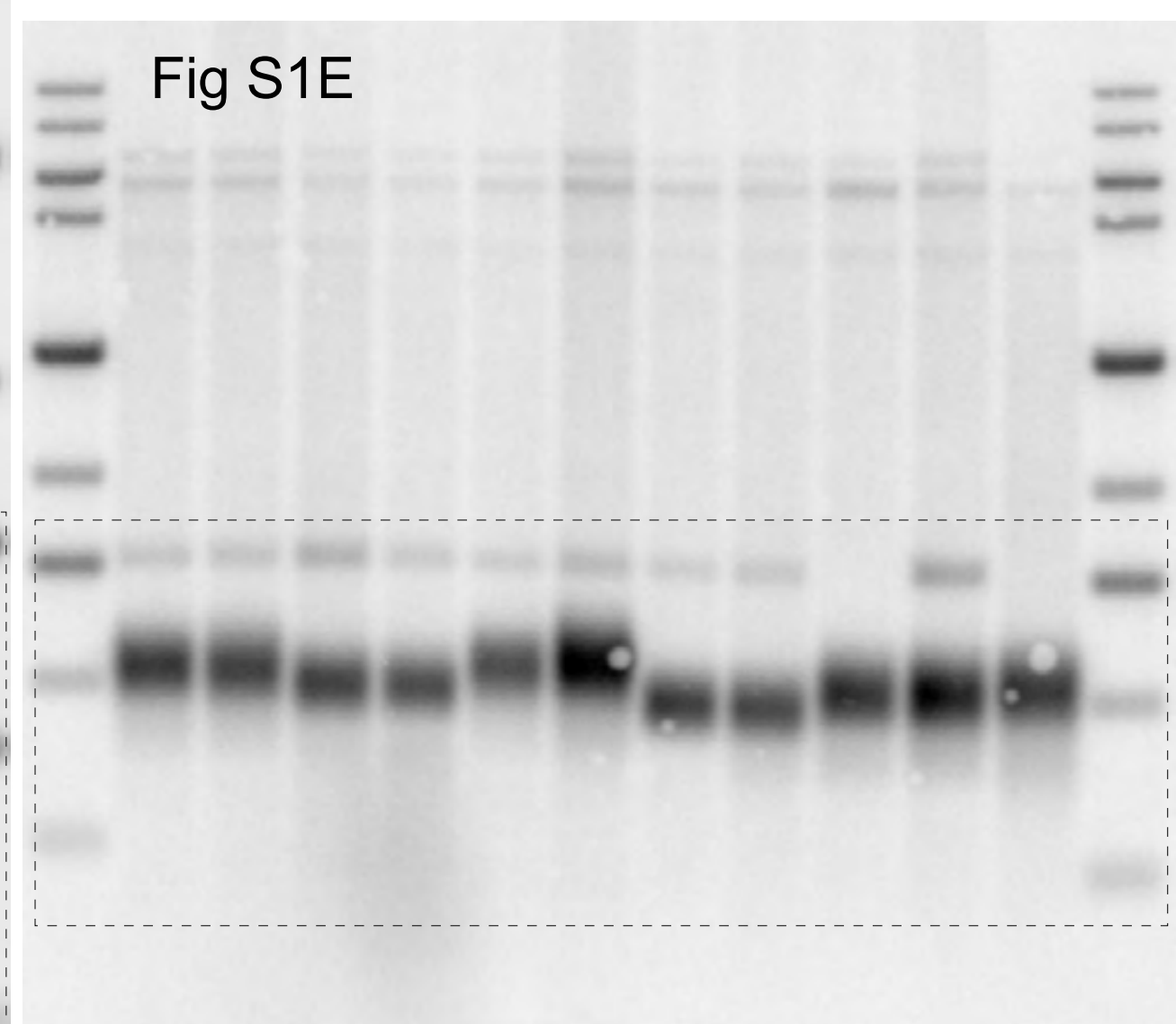

Fig S2D

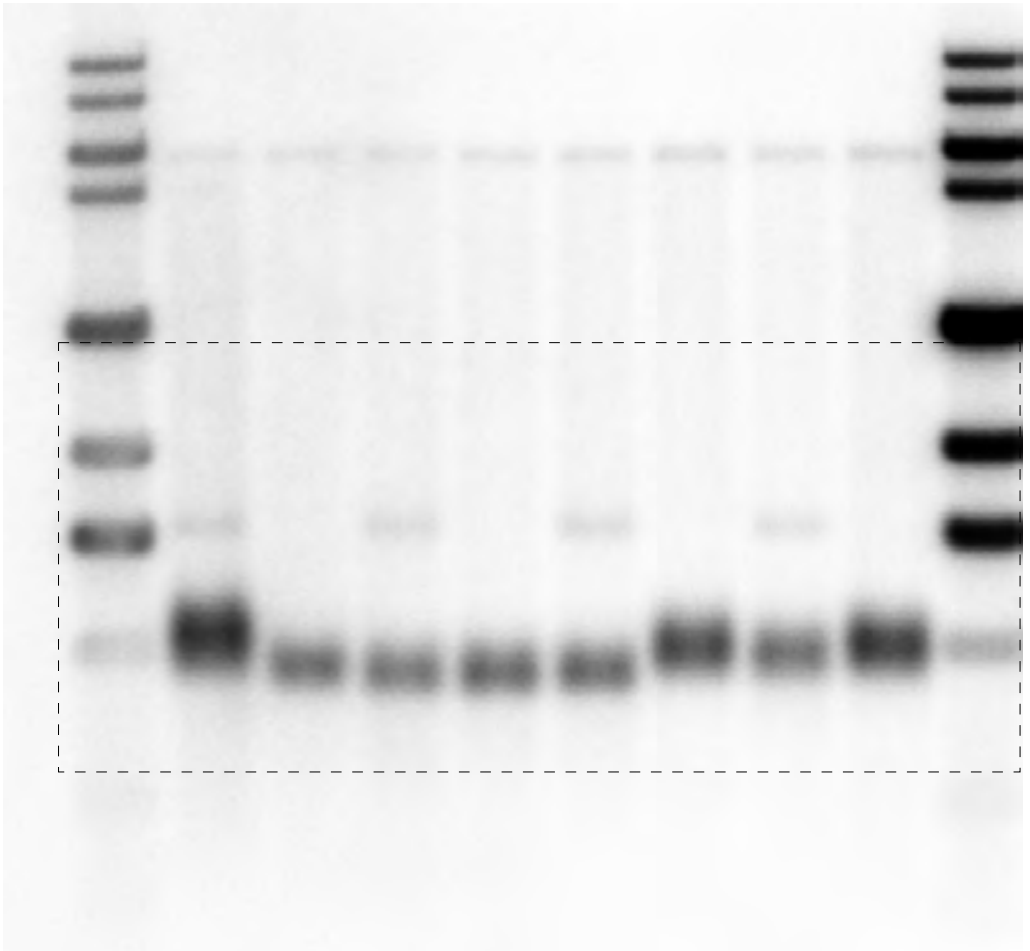

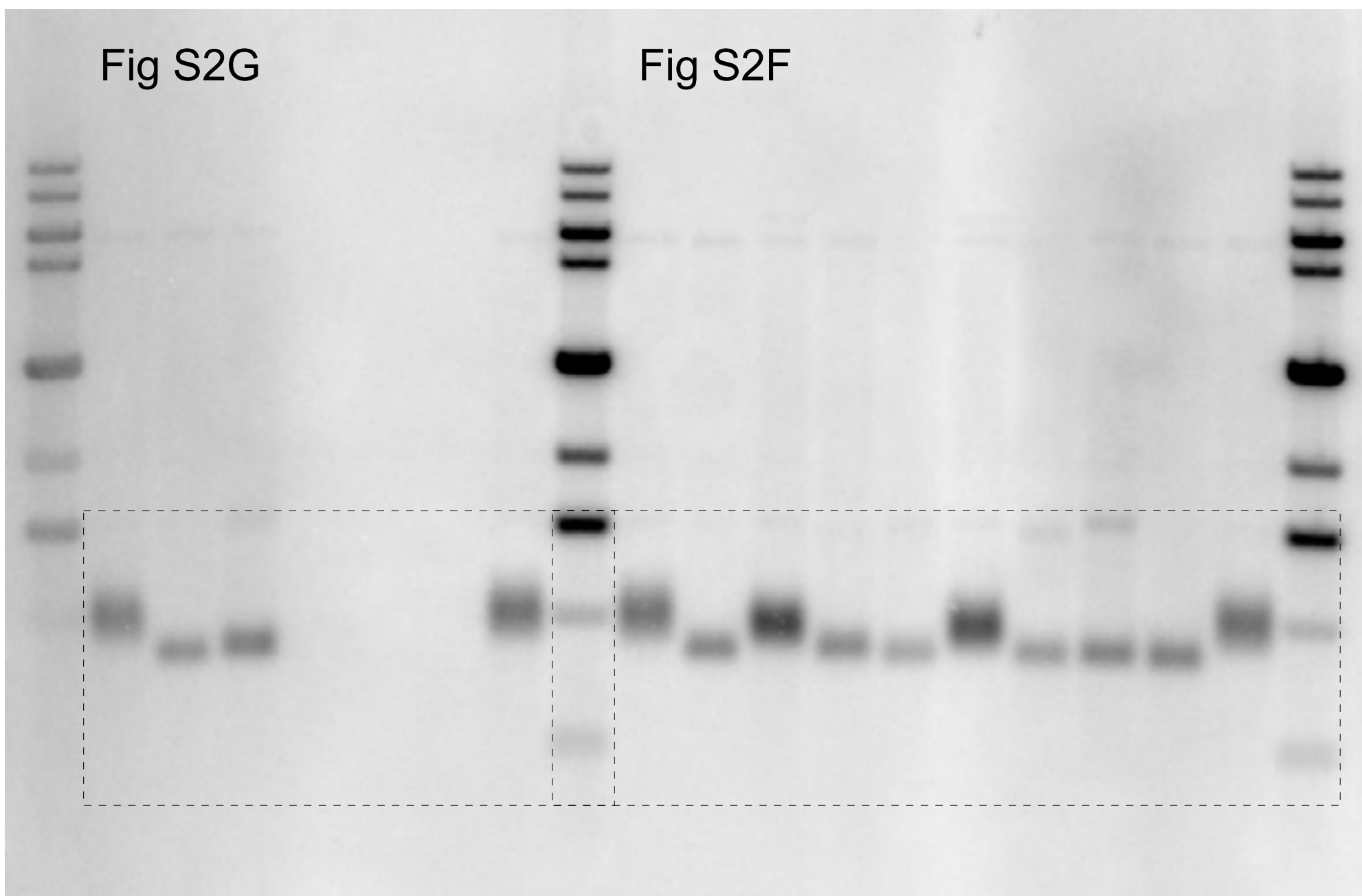

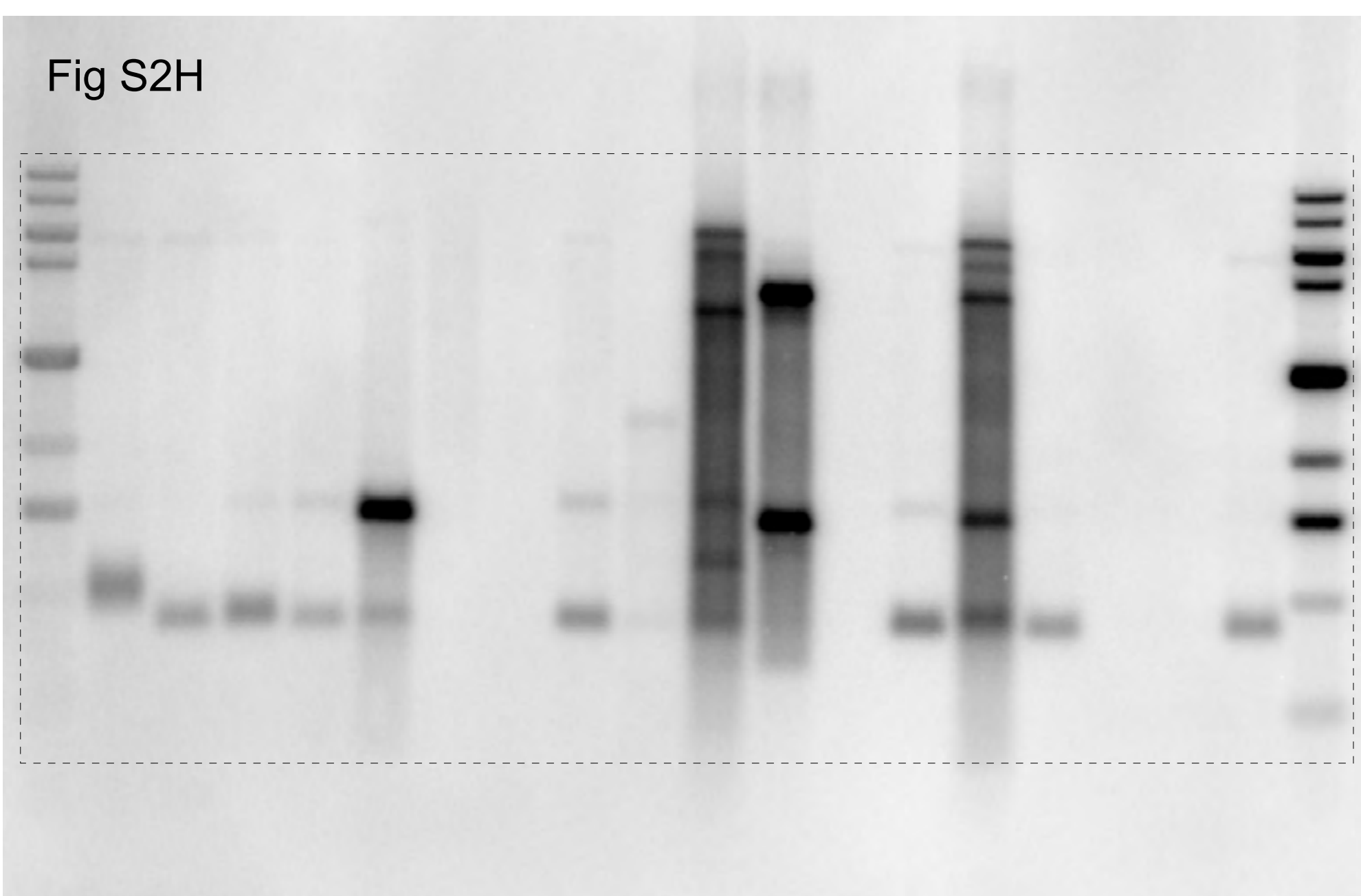

Fig S2C

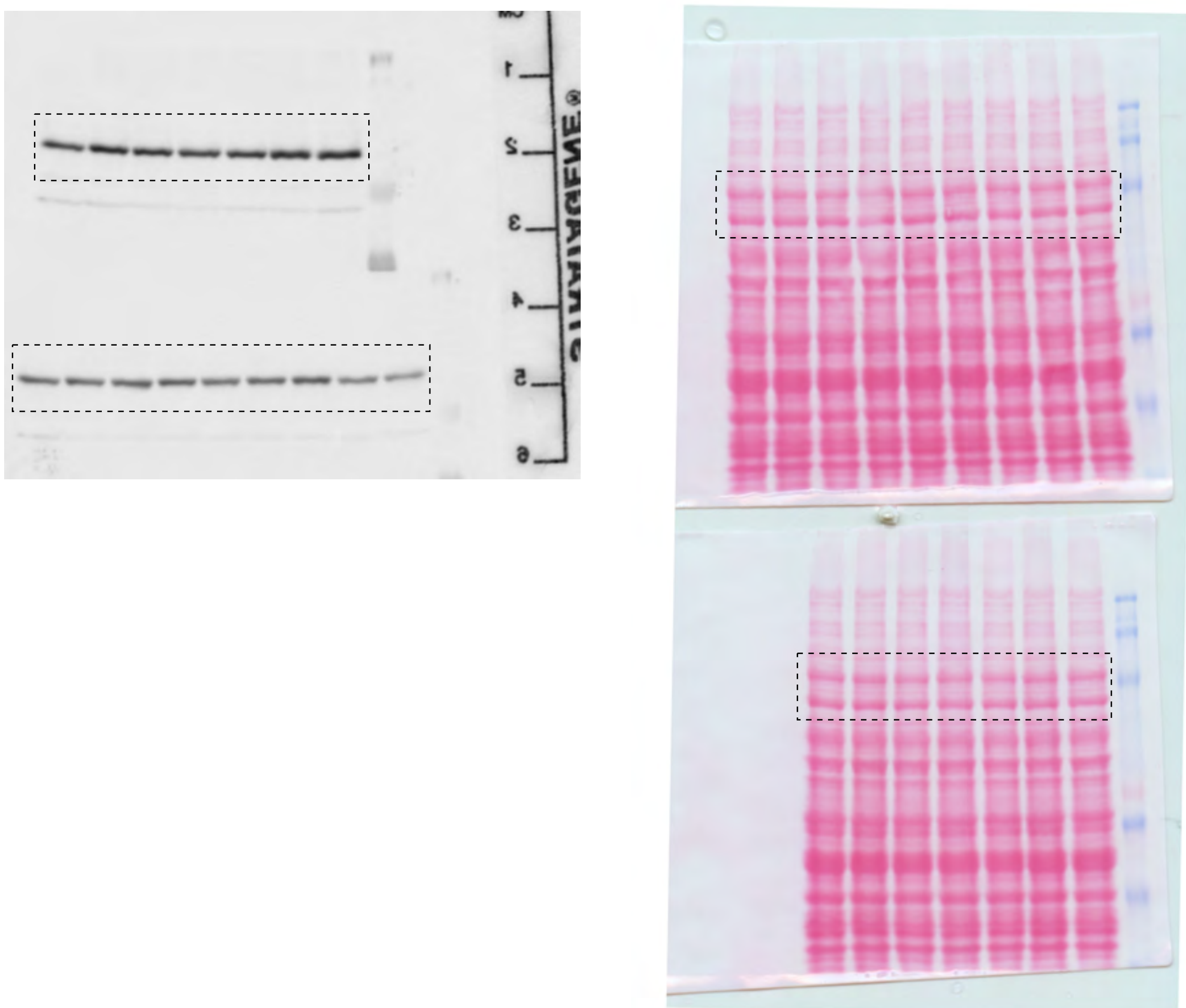

Fig S4A

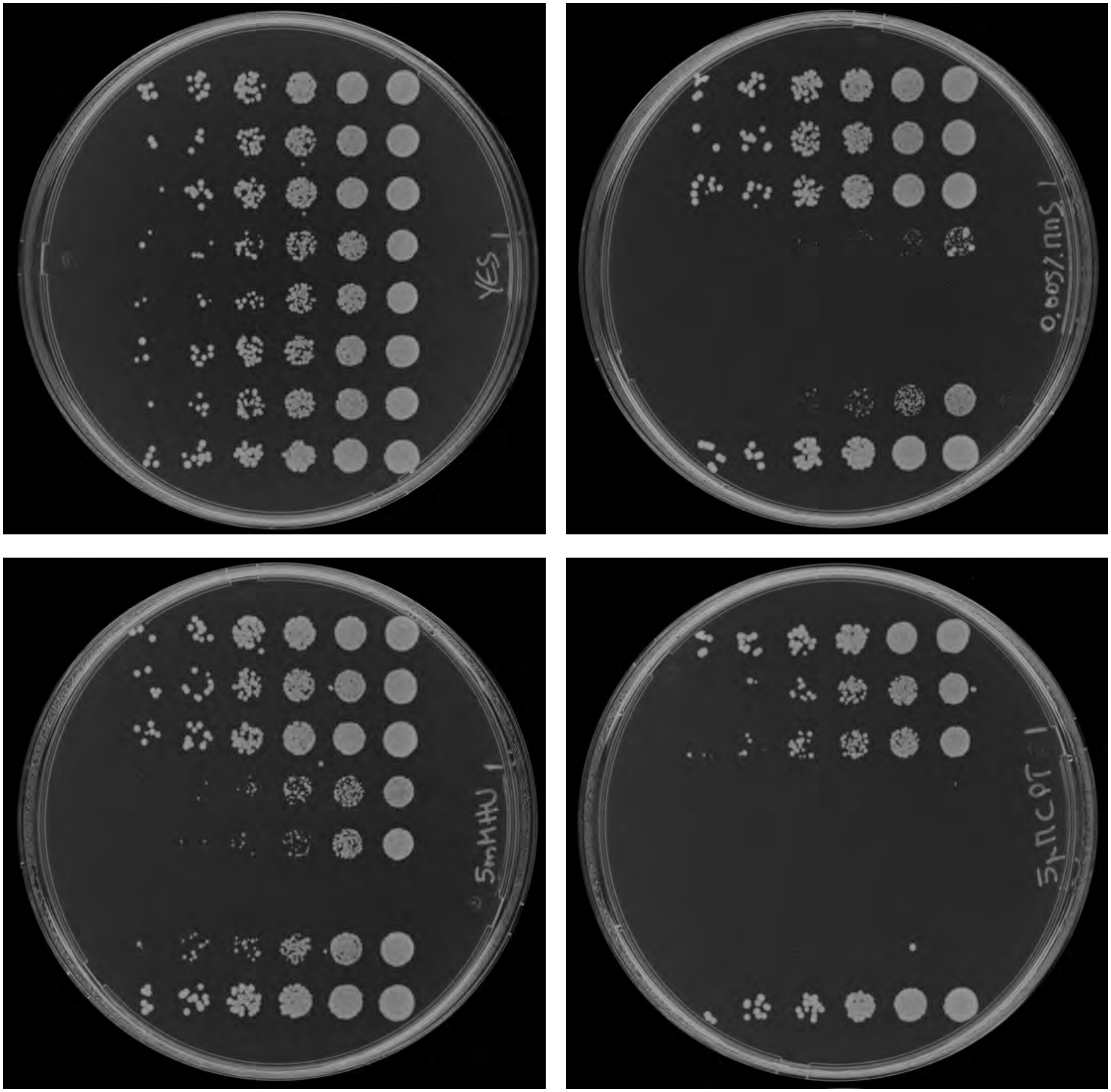

Fig S4B

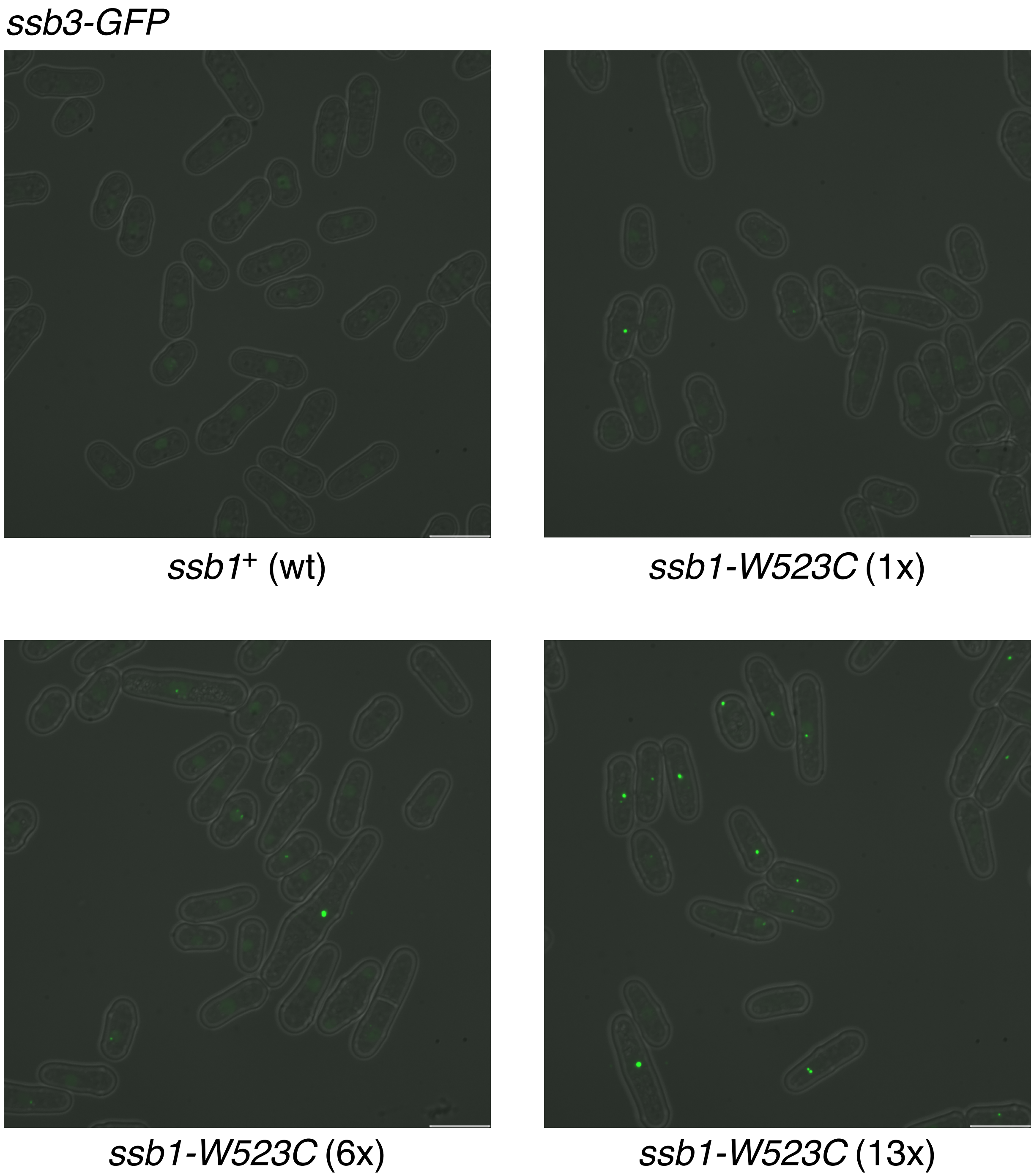

Fig S5B

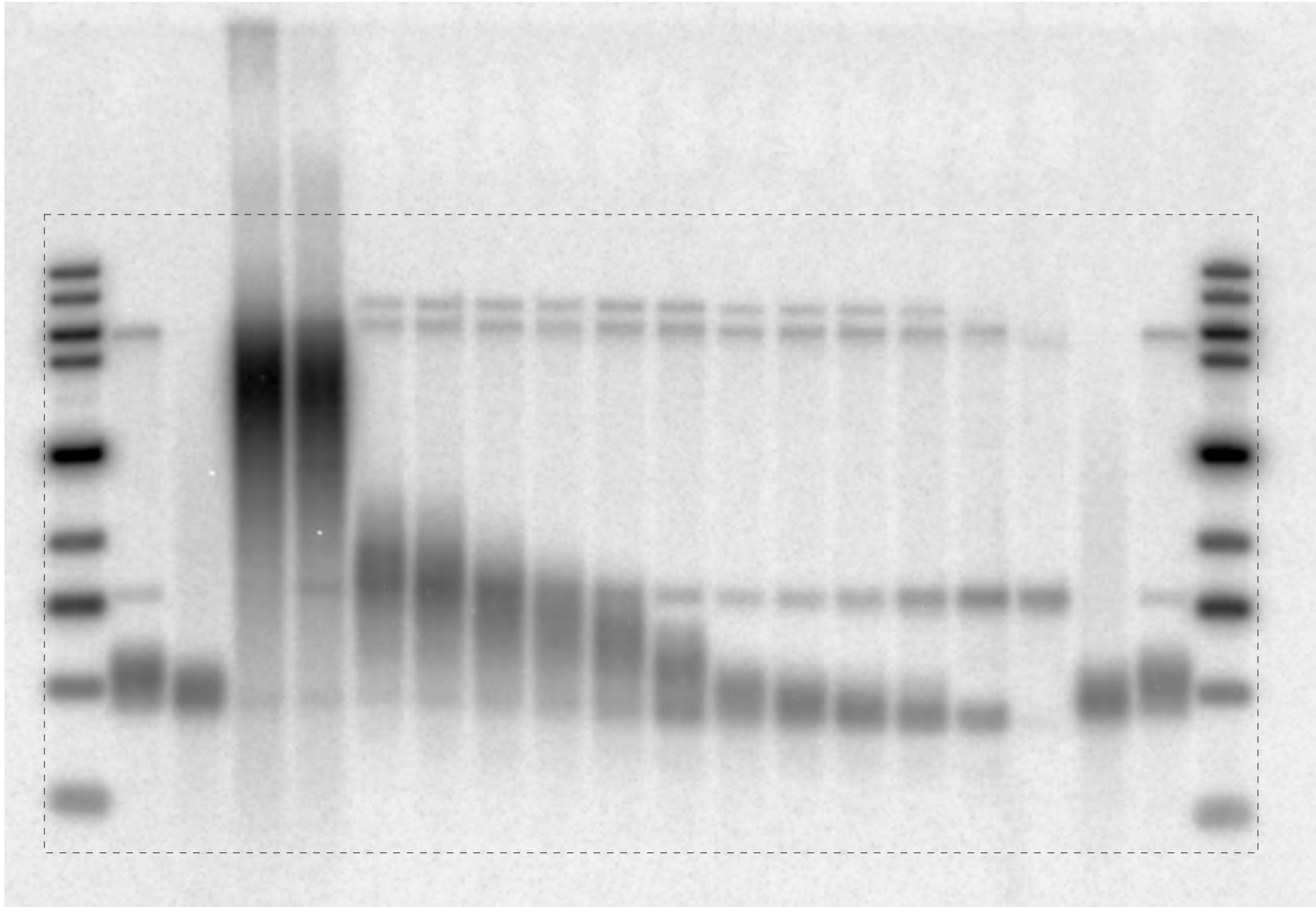

Fig S5C

Fig S5D

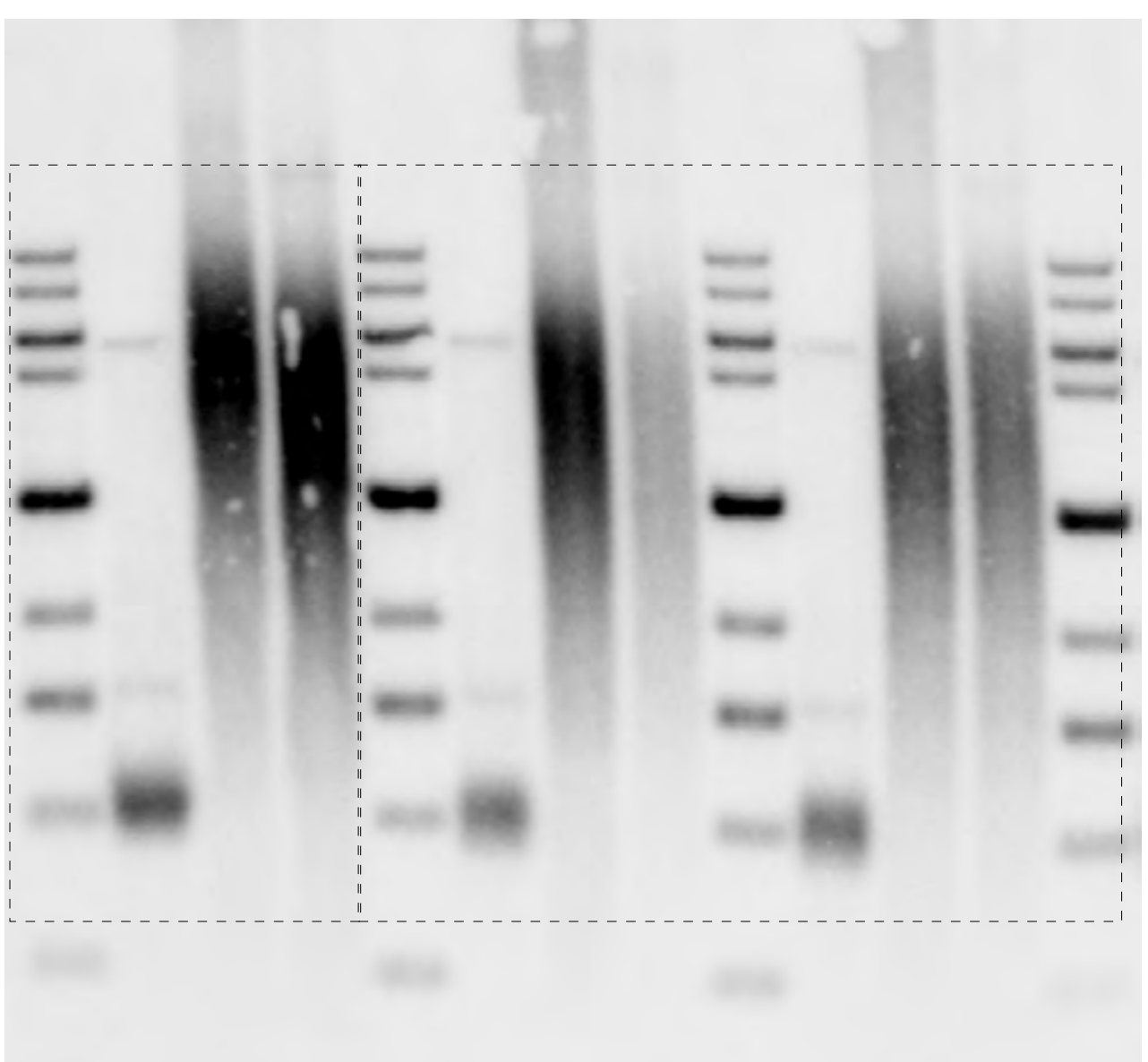

Fig S5E

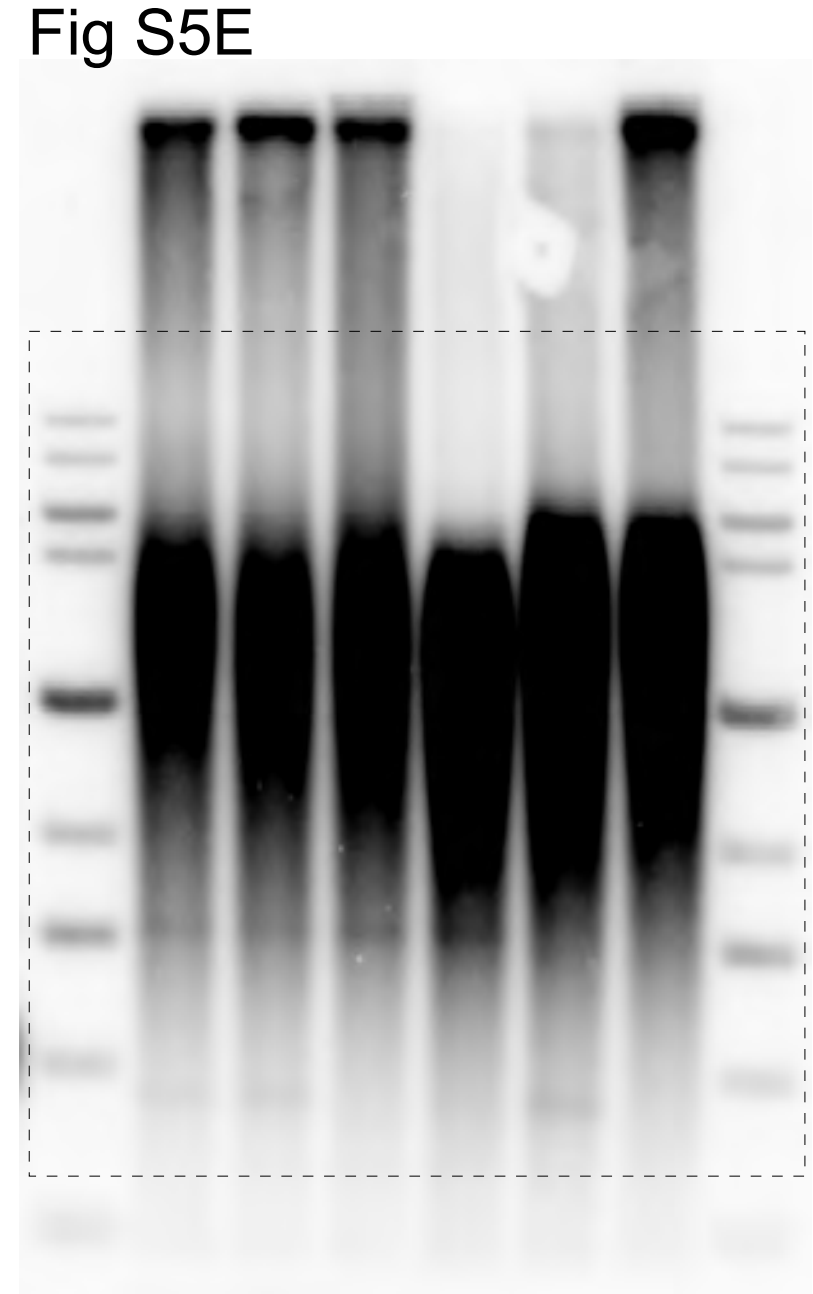

Fig S7B

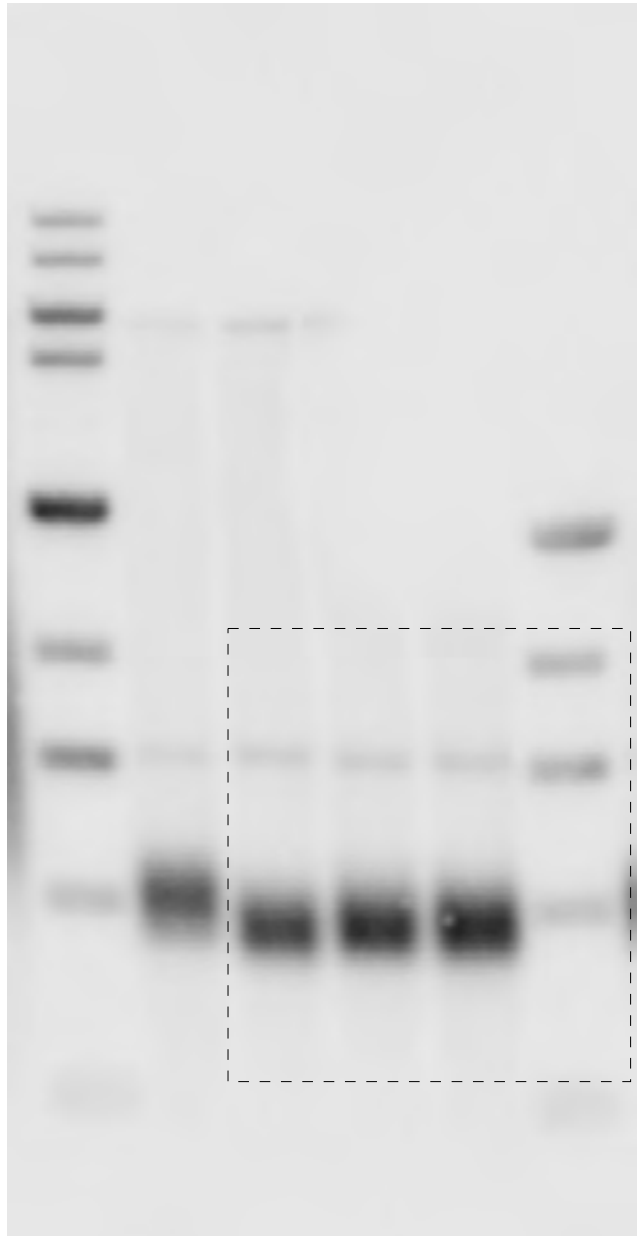

Fig S7C

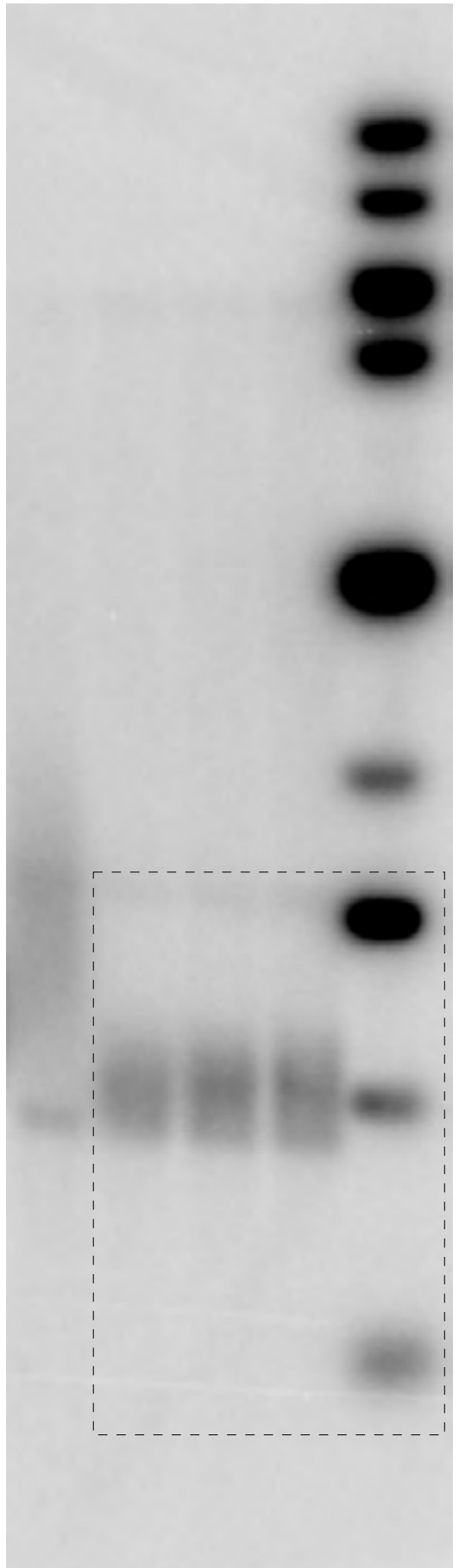

Fig S8A

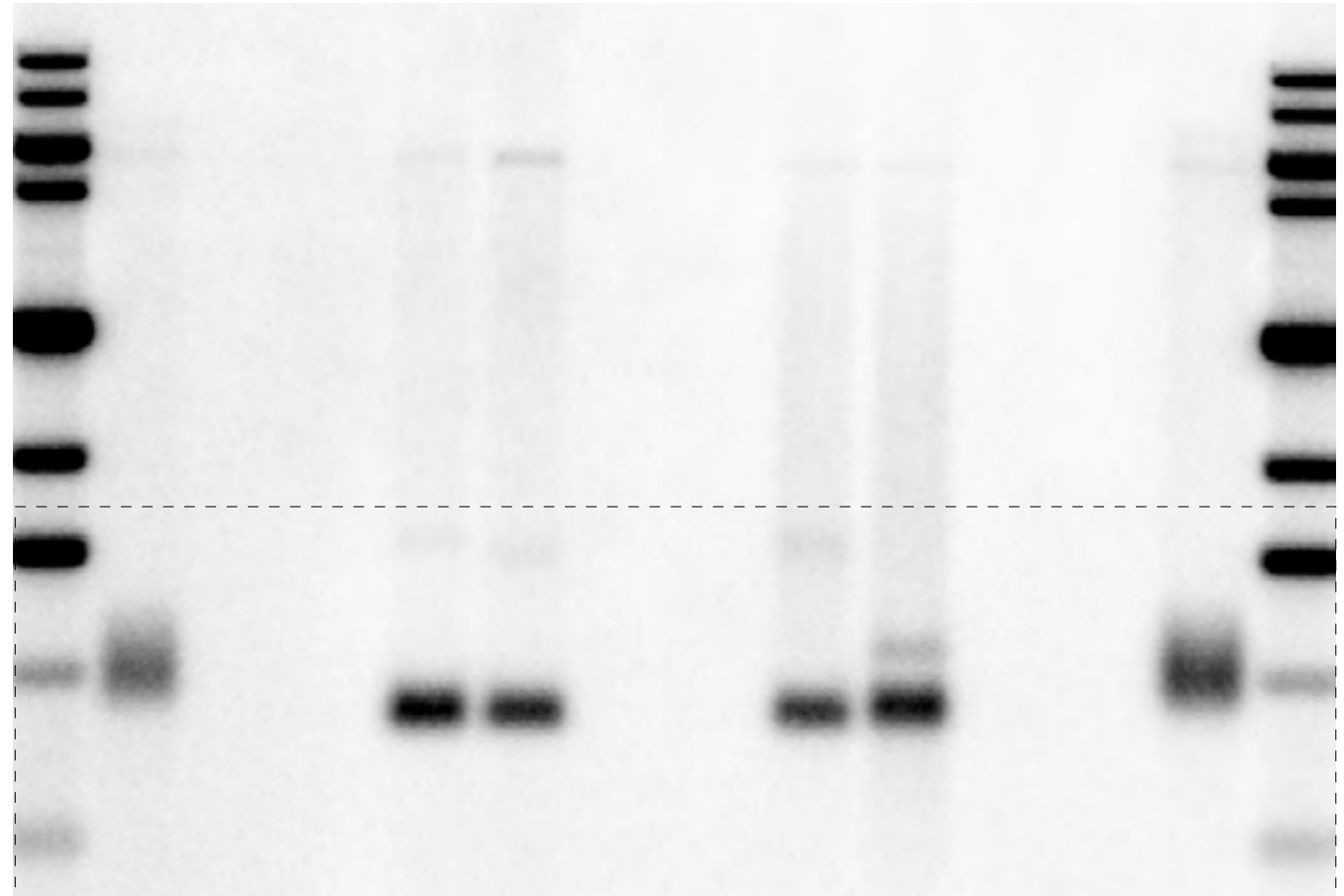

Fig S8B

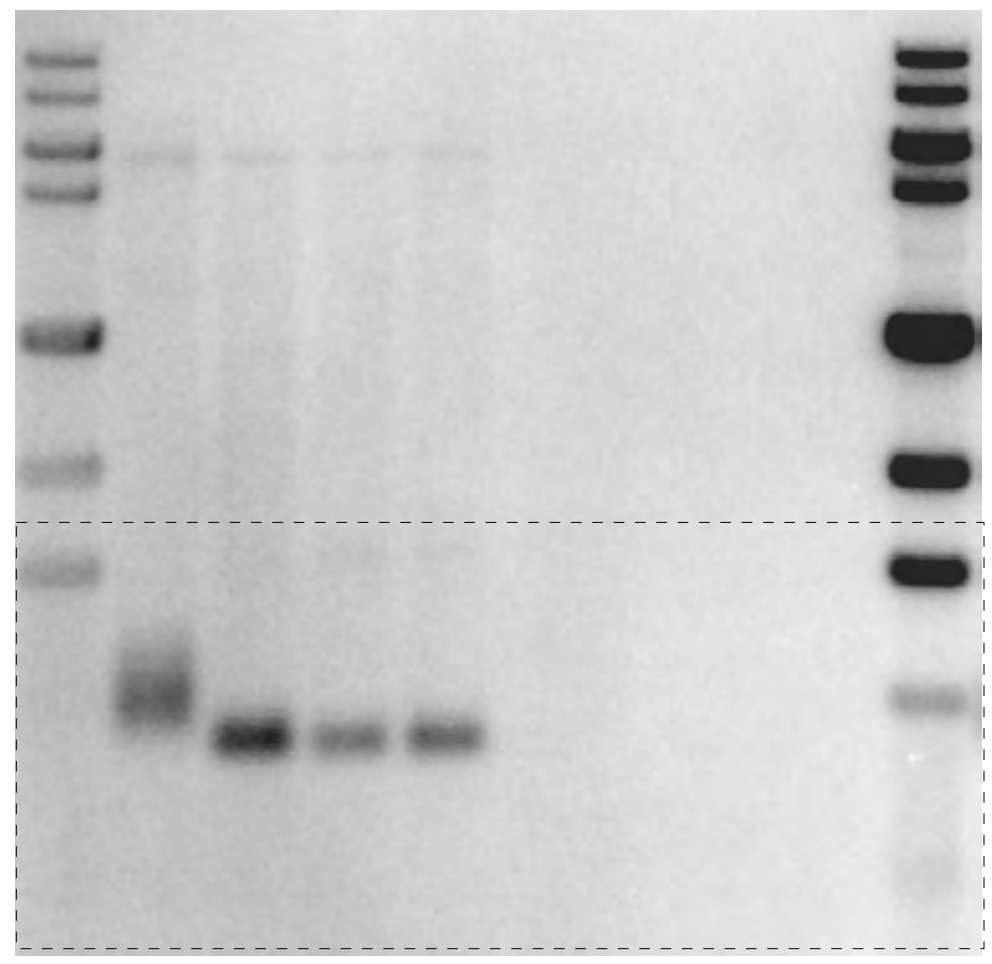

Fig S8C

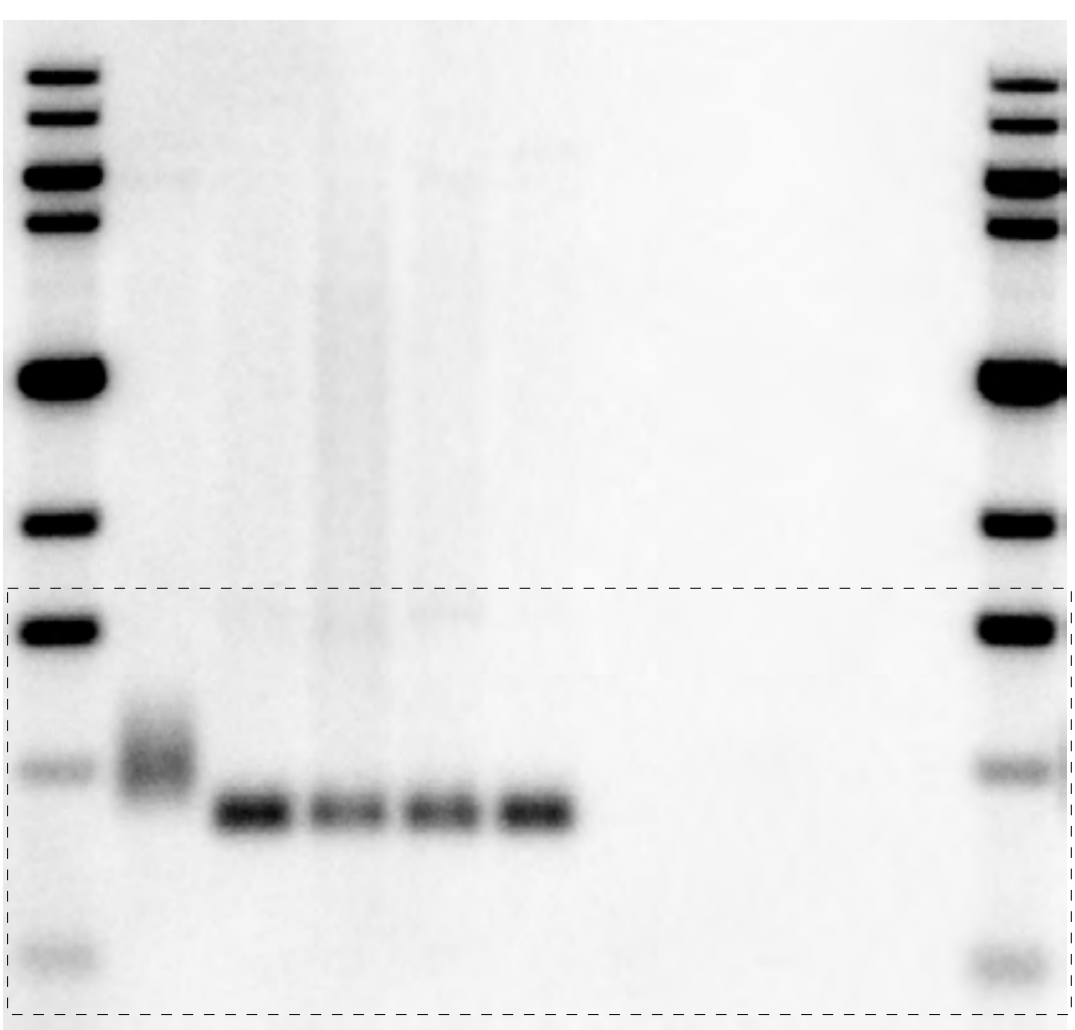

Fig S8D

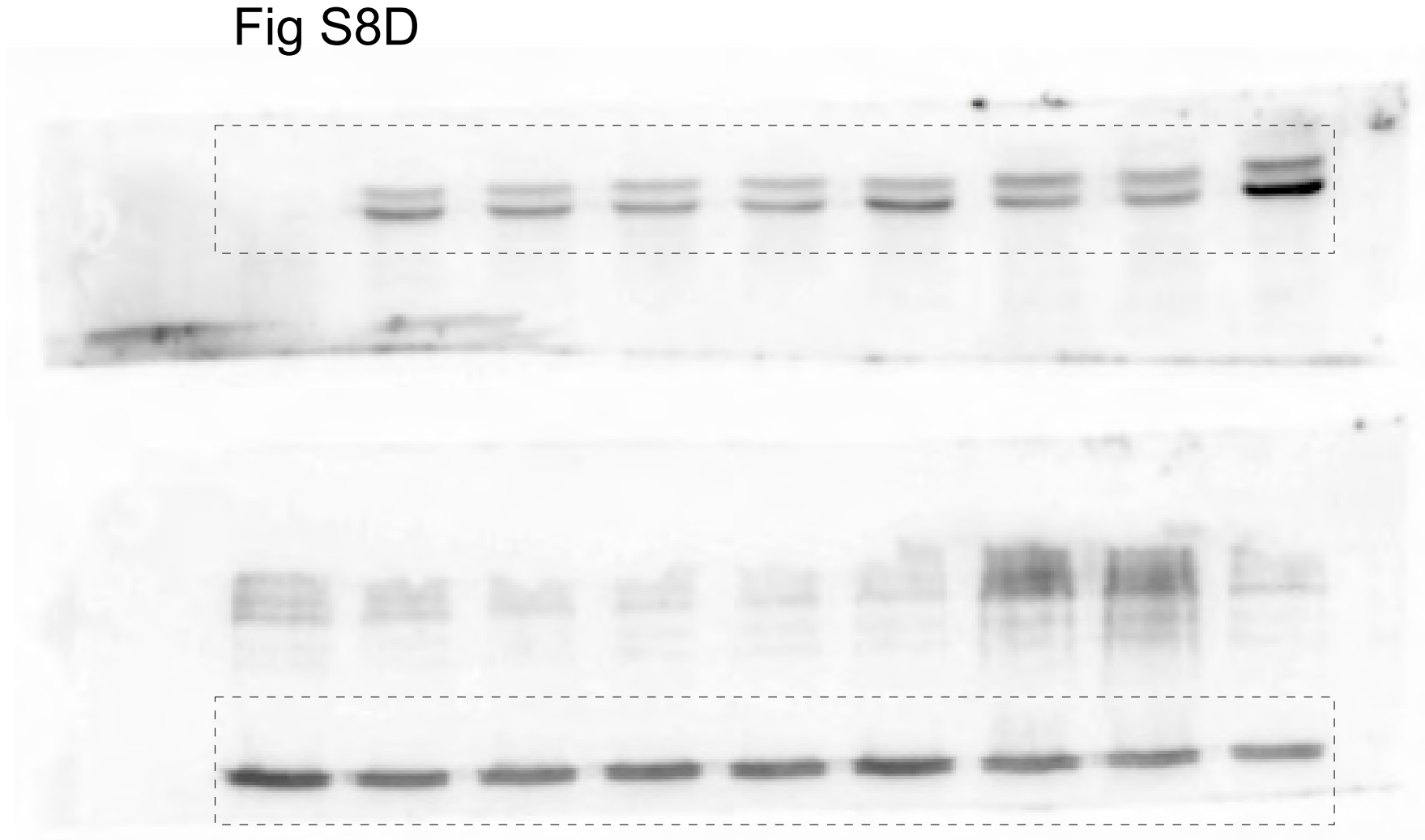

Fig S8G

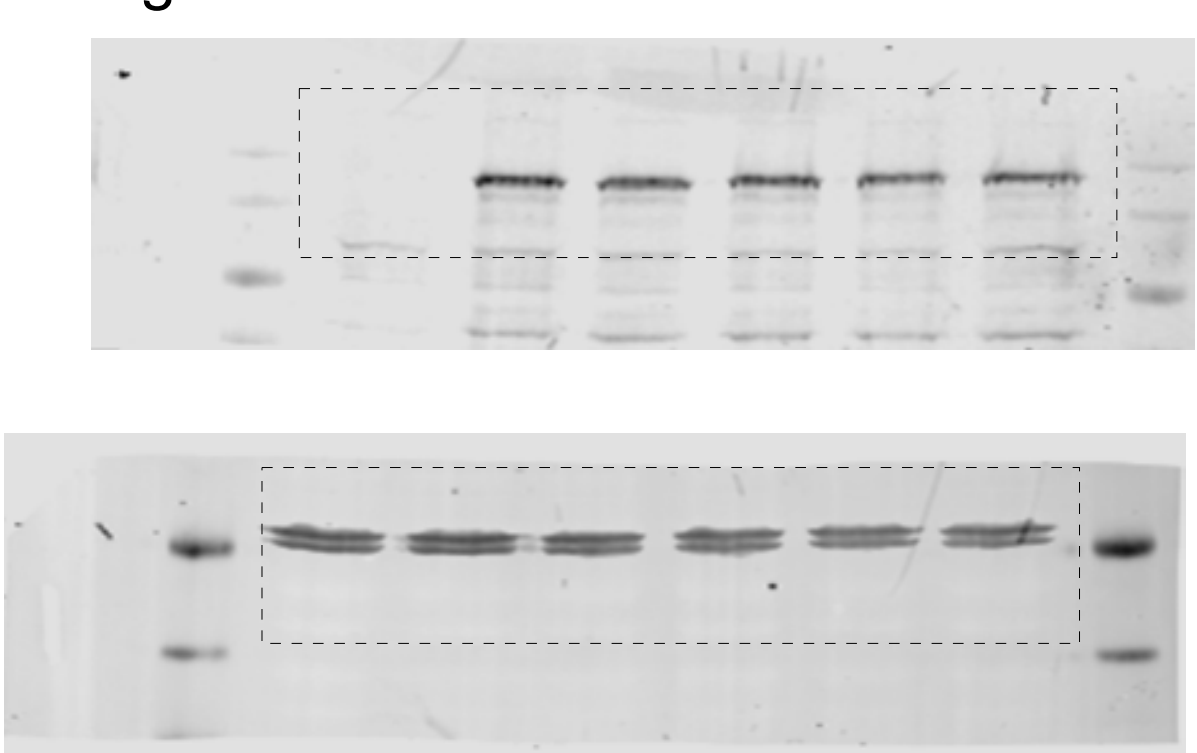

Fig S8E

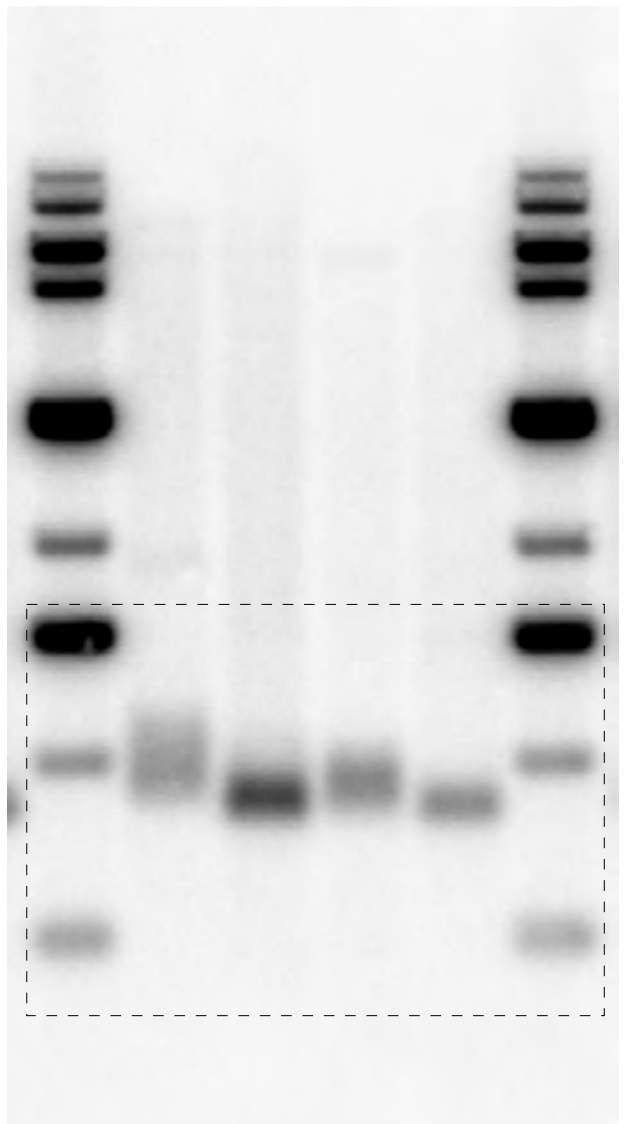

Fig S8F (left)

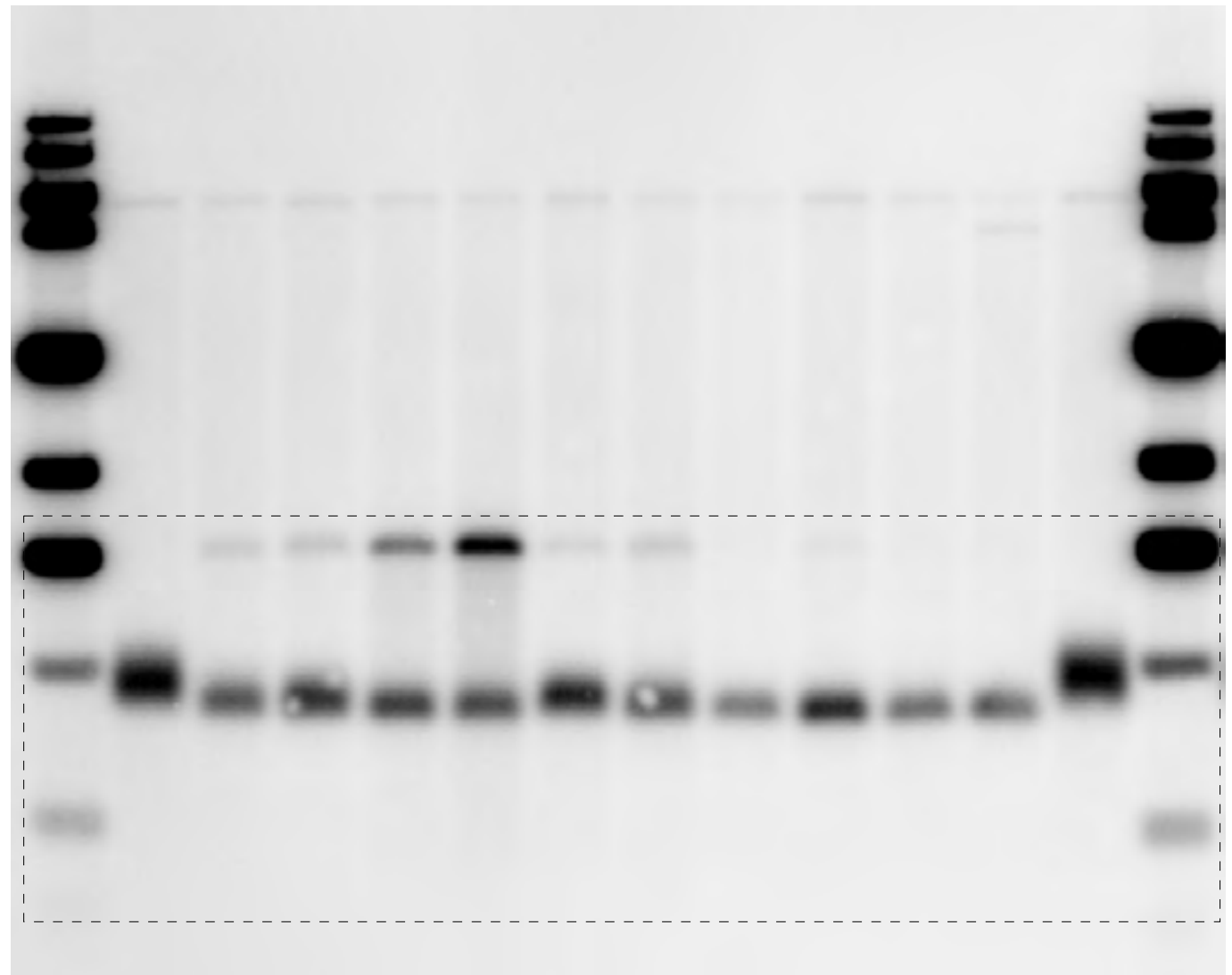

Fig S8F (right)

Fig S8H (right)

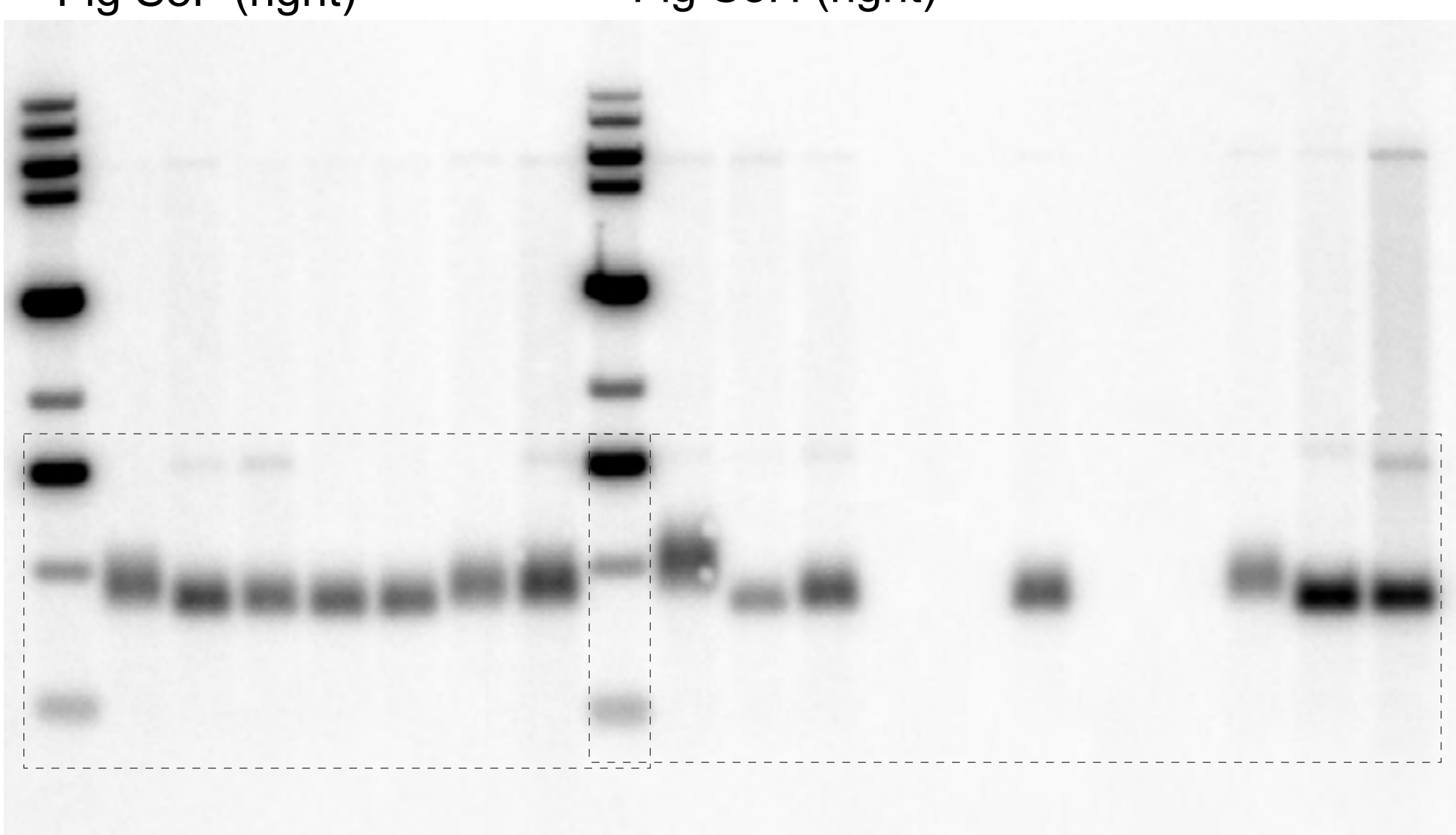

Fig S8H (left)

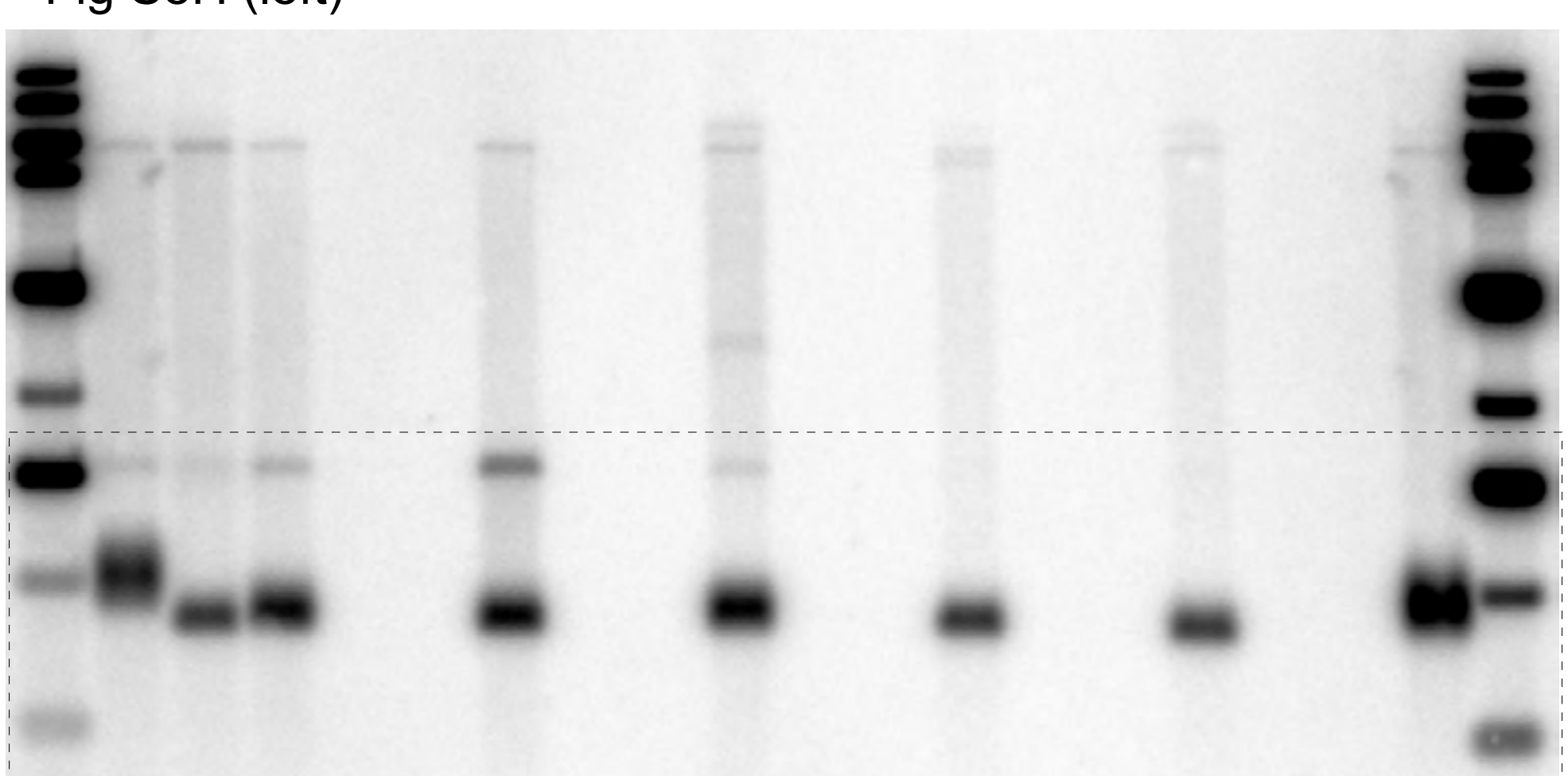

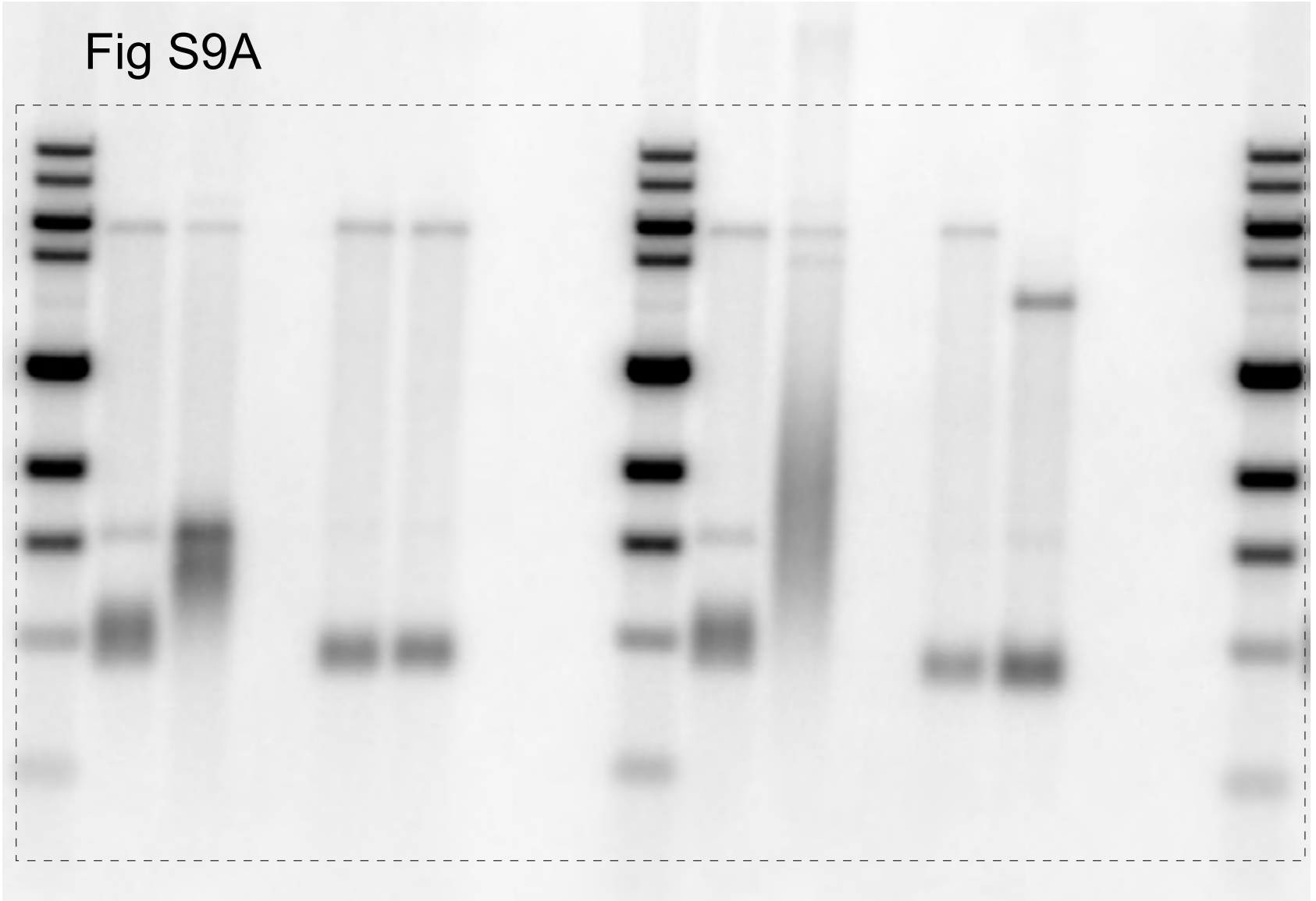
Fig S9A

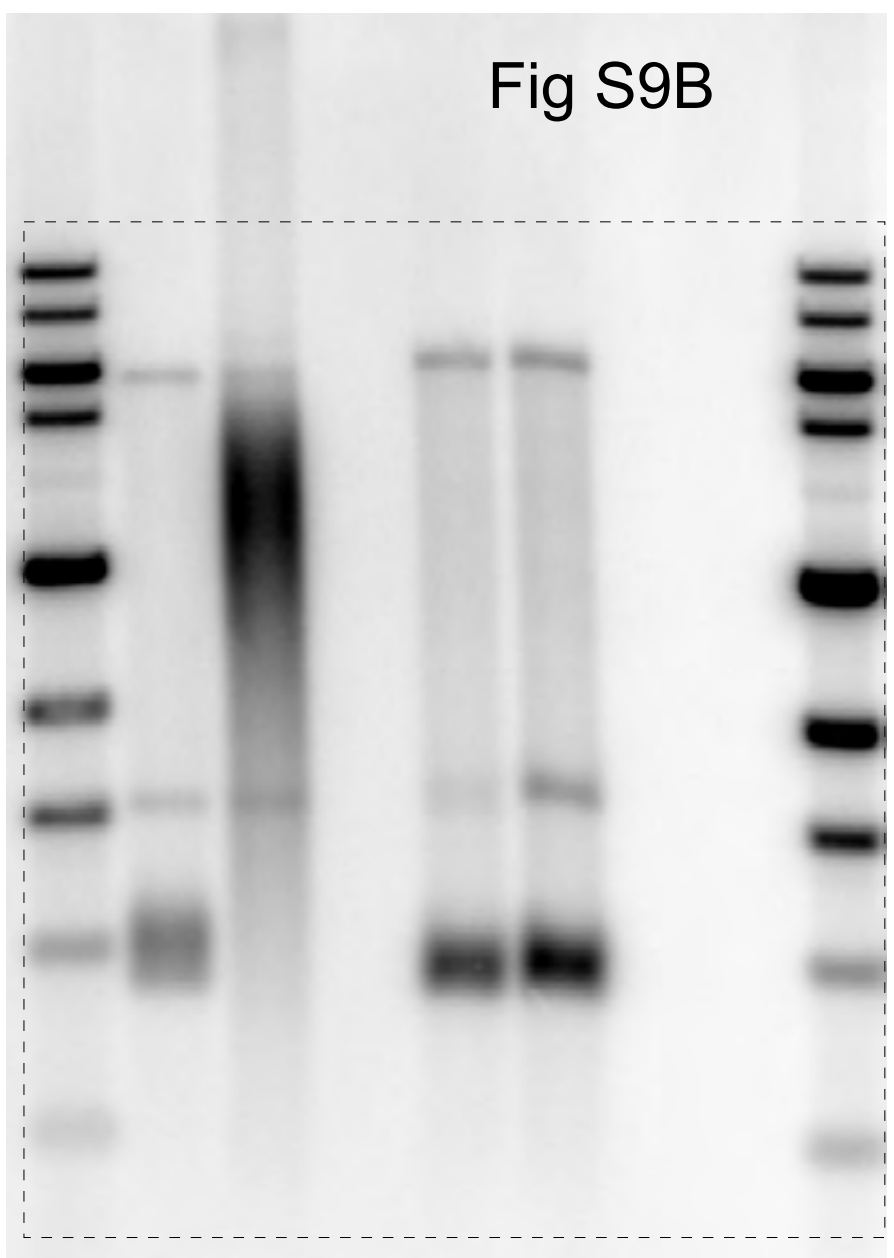
Fig S9B

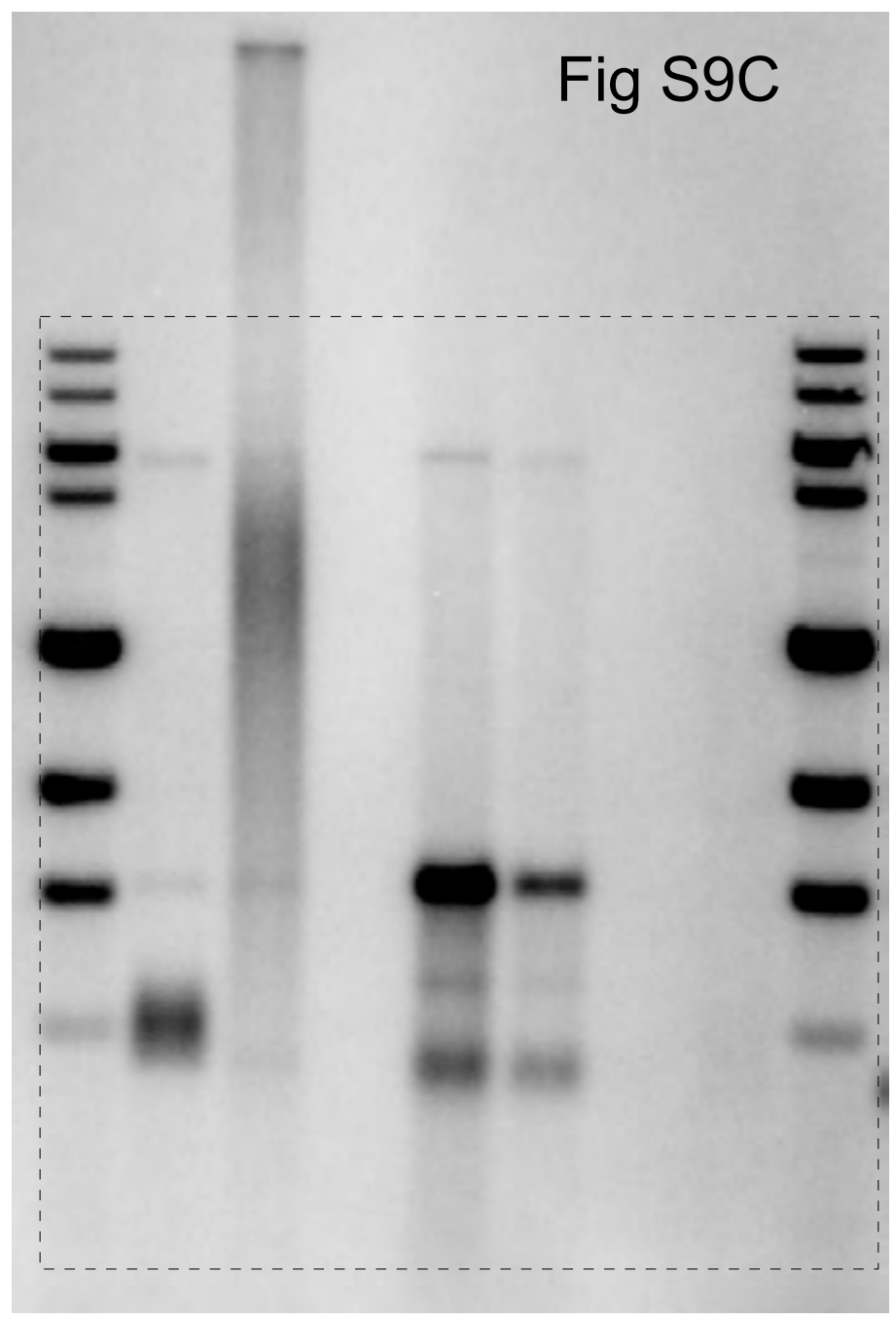
Fig S9C

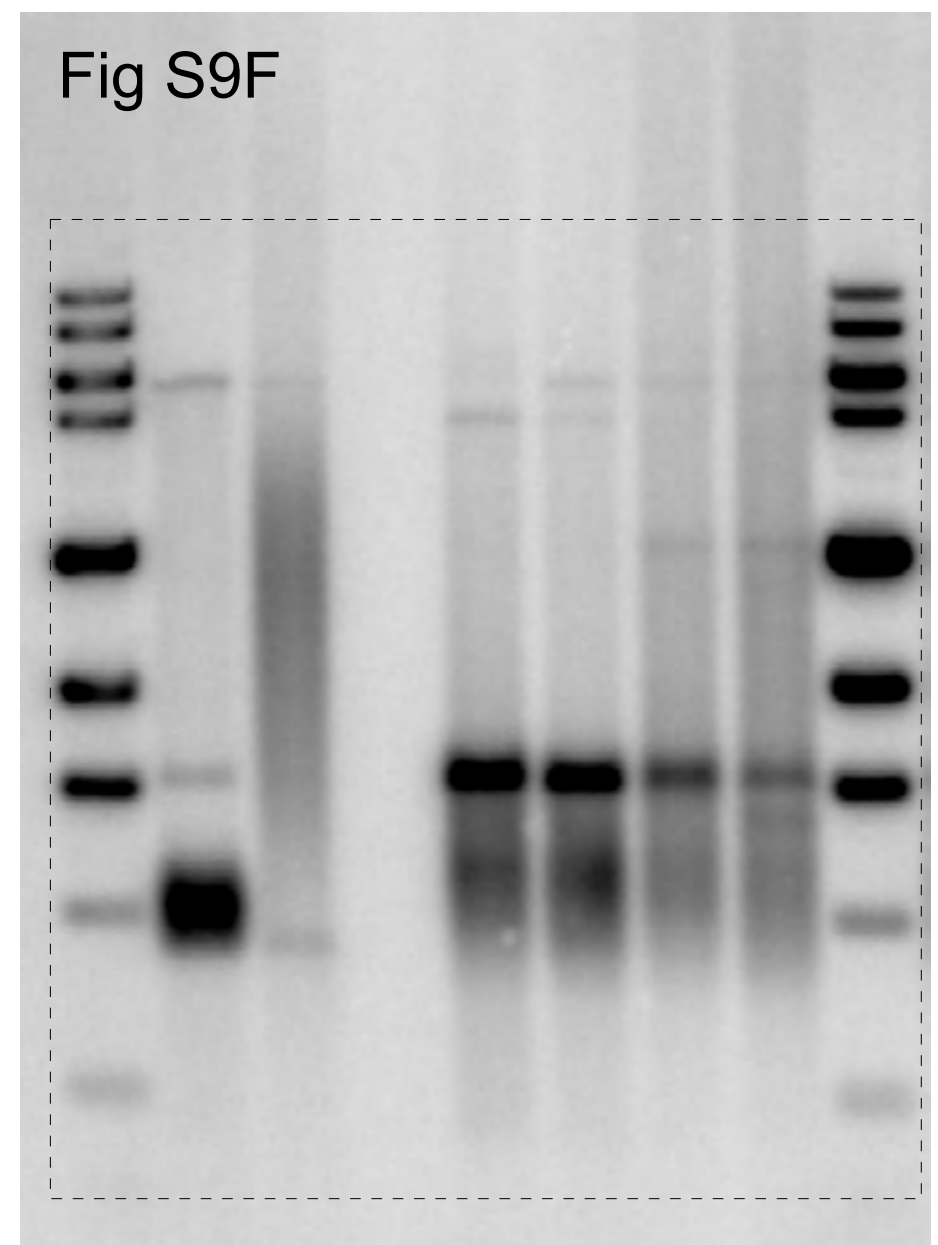
Fig S9F

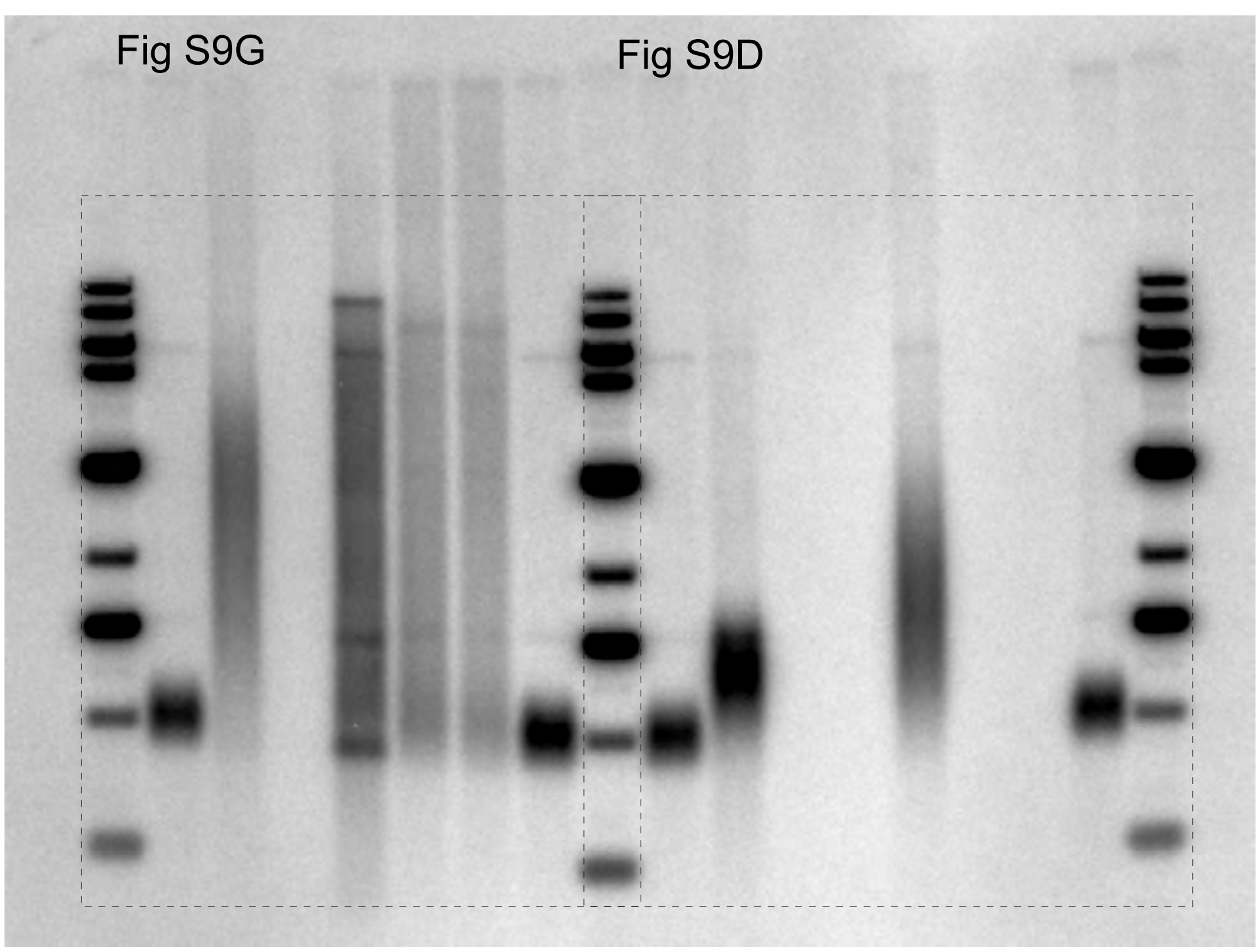
Fig S9G Fig S9D

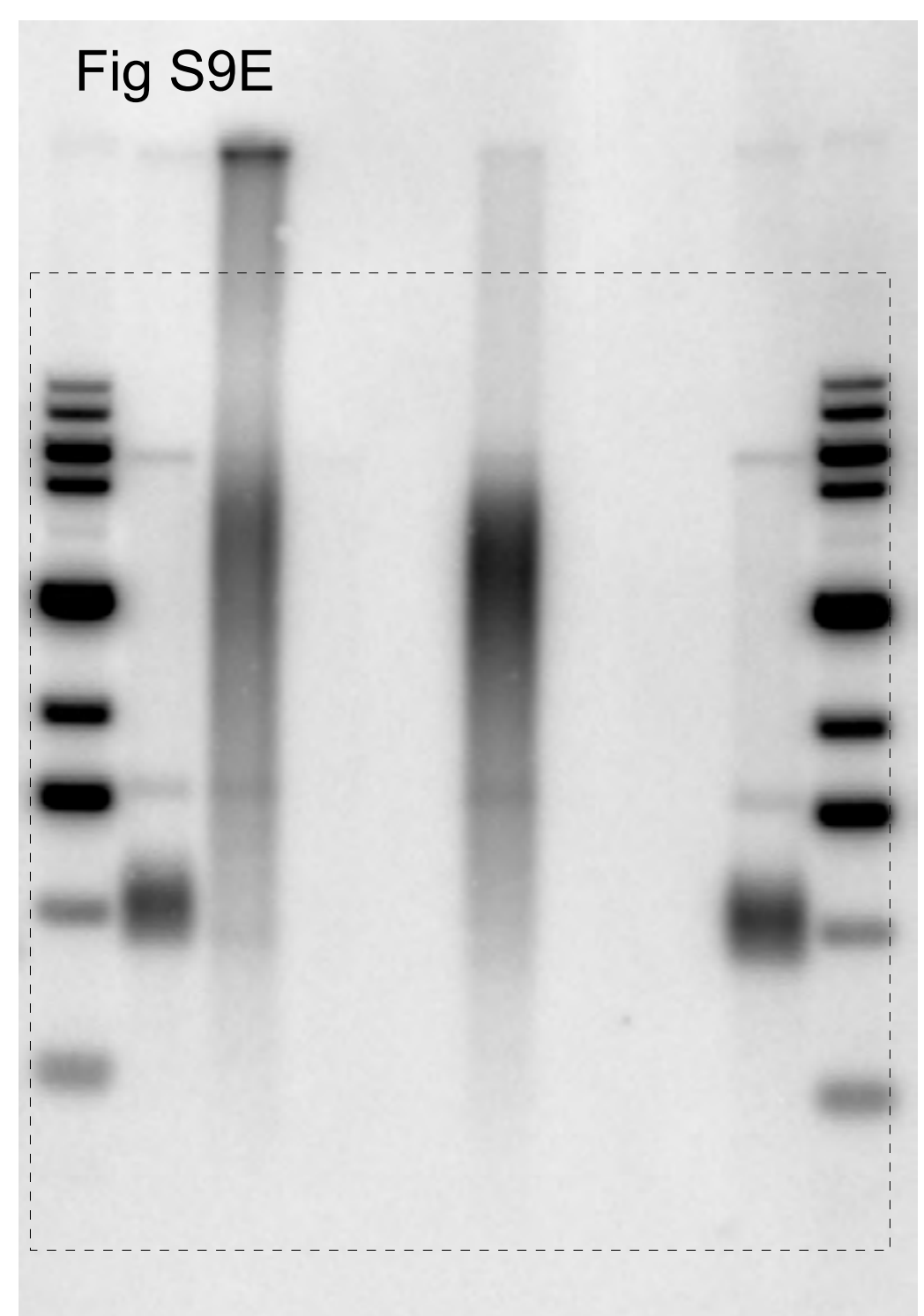
Fig S9E

Fig S10

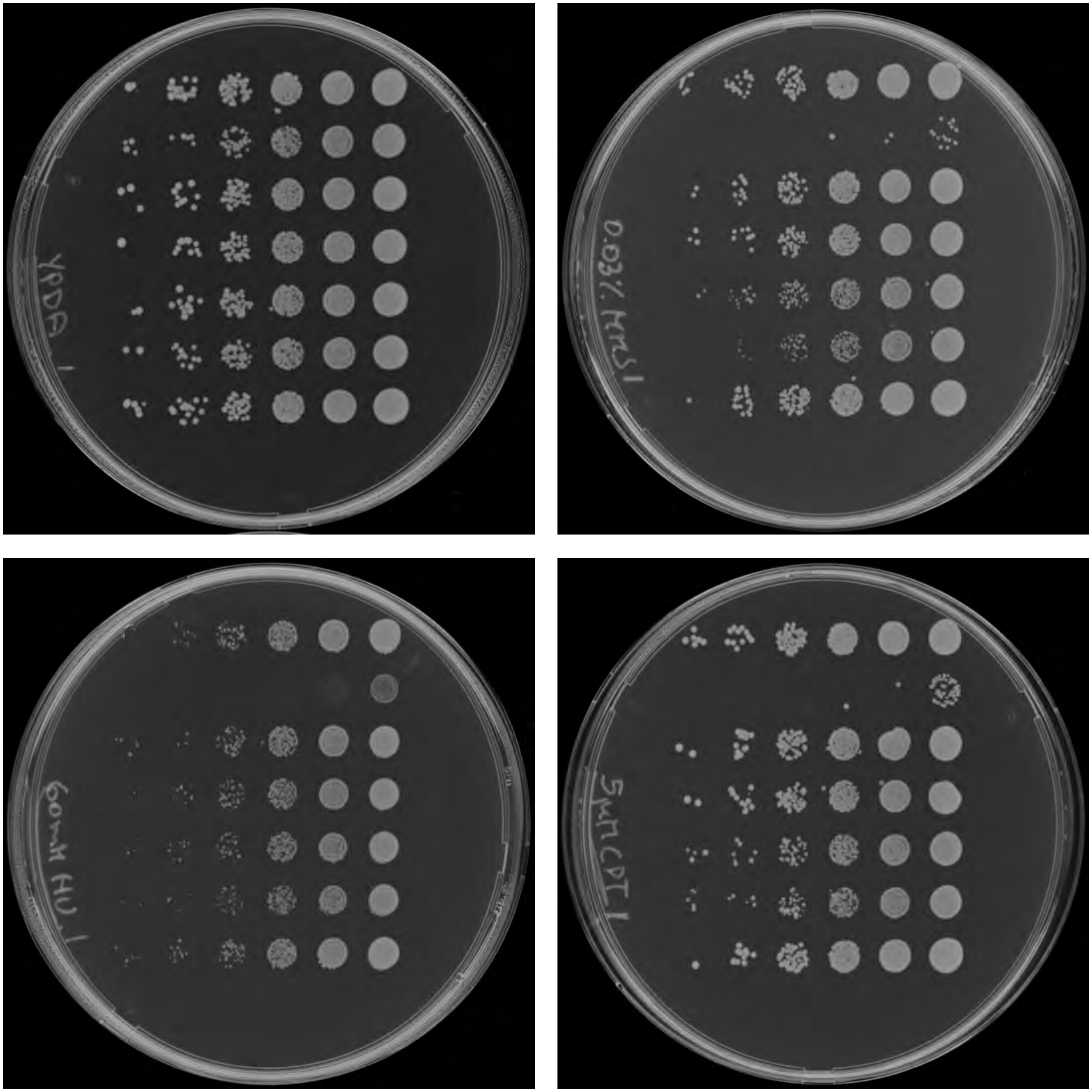

Supplement: Supplementary file 2 [file media-4.pdf]
